# *Polygonati Rhizoma* Prevents Glucocorticoid‐Induced Growth Inhibition of Muscle via Promoting Muscle Angiogenesis Through Deoxycholic Acid

**DOI:** 10.1002/jcsm.13853

**Published:** 2025-06-16

**Authors:** Shiyi Shi, Rui Li, Yanxu Han, Jiahao Xie, Shaoshuai Wang, Jie Liu, Fangyan Wan, Gaifeng Hou, Zuhong Liu, Xiaobo Sun, Bo Zuo, Zhihao Jia, Zhinan Mei, Tongxing Song

**Affiliations:** ^1^ College of Animal Science and Technology Huazhong Agricultural University Wuhan China; ^2^ Institute of Subtropical Agriculture Chinese Academy of Sciences Changsha China; ^3^ Institute of Animal Husbandry and Veterinary Sciences Wuhan Academy of Agricultural Sciences Wuhan China; ^4^ Academy of Chinese Medical Sciences Henan University of Chinese Medicine Zhengzhou China; ^5^ Cambridge‐Suda Genomic Resource Center, Suzhou Medical College Soochow University Suzhou China; ^6^ College of Plant Science and Technology Huazhong Agricultural University Wuhan China

**Keywords:** angiogenesis, deoxycholic acid, fructose, *Polygonati Rhizoma*, skeletal muscle

## Abstract

**Background:**

Glucocorticoids are commonly used in clinical treatments but can cause muscle growth inhibition and weakness at high doses. The mechanisms and treatments for glucocorticoid‐induced muscle growth inhibition remain poorly understood. This study aims to investigate the anti‐atrophic effects of *Polygonati Rhizoma* (PR) and a mixture of low‐dose fructose and glucose (MFG, an active component mimic in PR) on skeletal muscle.

**Methods:**

Male C57BL/6 mice (3‐week‐old, *n* = 8) were gavaged with aqueous extract of PR (AEPR). MFG was used to gavage normal male C57BL/6 mice (3‐week‐old, *n* = 10) and male C57BL/6 mice with dexamethasone (DEX)‐induced muscle growth inhibition (3‐week‐old, *n* = 7). After 2 weeks of gavage, the body weight and muscle mass of the mice were measured. Intestinal content was collected, the concentration of deoxycholic acid (DCA) was analysed and gut microbiota changes were assessed through 16S rRNA gene sequencing. Muscle angiogenesis was examined through the expression of vascular endothelial growth factors (VEGFs), focusing on the DCA‐activated TGR5/cAMP/PKA/pCREB pathway.

**Results:**

AEPR significantly increased the body weight (22.90 ± 0.90 vs. 21.83 ± 0.87 g, *p < 0.05) and grip strength (1.32 ± 0.11 vs. 1.04 ± 0.12 N, ***p < 0.001) of mice. MFG (0.5 g/kg body weight) also significantly elevated the body weight (21.44 ± 0.71 vs. 20.14 ± 0.82 g, **p < 0.01) and muscle mass (0.37 ± 0.018 vs. 0.33 ± 0.035 g, **p < 0.01) of mice. In the DEX group, MFG restored the DCA level (log_2_[intensity]) in intestinal content (25.41 ± 1.64 vs. 22.69 ± 0.74, *p < 0.05) and increased the abundance of *Collinsella aerofaciens* as measured by DNA concentration (0.80 ± 0.64 vs. 0.24 ± 0.09 pg/μL, *p* = 0.096). Mechanistically, MFG upregulated VEGFs expression and promoted muscle angiogenesis via the TGR5/cAMP/PKA/pCREB pathway.

**Conclusions:**

This study demonstrates that AEPR and its active component mimic MFG can promote muscle growth and MFG mitigates muscle growth inhibition by modulating gut microbiota and enhancing muscle angiogenesis. These findings suggest that fructose‐containing treatments are novel strategies to address skeletal muscle dysfunction.

## 1 Introduction

Glucocorticoids are commonly used to treat a vast array of chronic inflammatory diseases, such as systemic lupus erythematosus, sarcoidosis, rheumatoid arthritis and bronchial asthma [1]. However, administration of glucocorticoids at high doses can cause muscular dysfunction in humans and animals [2, 3]. Glucocorticoids may inhibit protein synthesis and stimulate protein degradation in skeletal muscle [4]. However, the mechanism by which glucocorticoids cause muscle growth inhibition remains poorly understood.

Skeletal muscle growth inhibition is characterized by several changes in muscle such as reduction in myofibre size, changes in fibre type or myosin isoforms and overall loss of cytoplasm, organelles and protein [5]. The growth, function and metabolism of skeletal muscle are regulated by various neural and humoral factors, as well as by vascular development and nutrient supply within the muscle [6]. Importantly, the reduction of capillaries in skeletal muscle can impede the transport of substrates, oxygen, hormones and nutrients, which can cause functional impairment and sarcopenia, a syndrome characterized by progressive loss of muscle mass and strength in older individuals [7, 8]. Thus, intramuscular angiogenesis is a basic and potential therapeutic target for alleviating muscle growth inhibition. The mechanisms of intramuscular angiogenesis are dynamically mediated by the coordinated interplay of multiple factors, including hypoxia, mechanical force and metabolic signalling, where the VEGF, Notch and angiopoietin systems constitute the core signalling pathways [9, 10].

PR, which is derived from the root of *Polygonatum cyrtonema* Hua., is a traditional Chinese herbal medicine with a wide range of pharmacological activities both in vivo and in vitro, such as anti‐ageing, anti‐hypoglycaemic, neuroprotective and neuroremodelling activities. Notably, the process of neuronal network formation is similar to the development of the vascular system [11]. It has been reported that PR can increase angiogenesis in skin tissues [12]. However, whether PR can promote angiogenesis in muscle tissues and subsequently enhance muscle development remains to be determined.

PR is rich in fructose [13]. Fructose and high‐fructose corn syrup (HFCS) have sparked controversy. HFCS, which comprises 42% or 52% fructose (dry basis) and a large amount of glucose [14], has been associated with increased risks of metabolic syndromes such as obesity and fatty acid liver diseases. Therefore, fructose has been predominantly labelled with negative effects [14]. However, fructose is canonically identified as a nutrient under normal physiological conditions. Interestingly, recent research has revealed that dietary fructose (25% HFCS in tap water) can increase intestinal villus length and enhance nutrient absorption in various mouse models [15]. Moreover, it has been found that although high doses of fructose (together with glucose at a 1: 1 ratio, > 1 g/kg body weight for gavage) may exceed the absorption and clearance ability of the intestine and result in spillover of fructose to the liver, about 90% of low‐dose fructose (0.25 g/kg) could be cleared by the intestine, indicating an intestinal first‐pass effect [16]. These findings suggest that low‐dose fructose may have a positive effect. However, it remains unclear how low‐dose fructose affects the gut microenvironment and thus may exert some alleviating effects on glucocorticoid‐induced skeletal muscle growth inhibition.

In this study, we investigated the effects of AEPR and its active component mimic MFG on the growth and glucocorticoid‐induced growth inhibition of mouse skeletal muscle. We found that MFG could ameliorate DEX‐induced skeletal muscle growth inhibition, probably through promoting the angiogenesis and biological function of muscle via the microbiota‐derived DCA‐induced TGR5/cAMP/PKA/pCREB axis. Taken together, our results reveal a novel mechanism for enhanced angiogenesis to alleviate muscle growth inhibition through manipulation of gut microbiota and suggest that AEPR and MFG may help to counteract glucocorticoid‐induced muscle growth inhibition in humans.

## 2 Methods

### 2.1 Animals

The C57BL/6 mice used in this study were obtained from Huazhong Agricultural University. All animal care, feeding and experimental procedures were conducted in accordance with the guidelines set forth by the National Research Council Guide for the Care and Use of Laboratory Animals and were approved by the Institutional Animal Care and Use Committee at Huazhong Agricultural University. The C57BL/6 mice were housed under standard conditions, with a temperature of 22 ± 1°C and access to a regular chow diet and water *ad libitum*.

### 2.2 Mouse Model of DEX‐Induced Muscle Growth Inhibition

Twenty‐one 3‐week‐old male C57BL/6 mice were randomly assigned to three groups, with each group consisting of seven mice (*n* = 7). The three groups were treated as follows. For the CON group, the mice received intraperitoneal injection of polyethylene glycol 400 (PEG400) solution (P815616, Macklin, China) with an equivalent volume to that of the DEX group; for the DEX group, the mice received intraperitoneal injection of DEX (10 mg/kg/day) for a duration of 2 weeks; for the DEX‐MFG group, the mice were intraperitoneally injected with DEX (10 mg/kg/day) for 2 weeks, followed by gavage with MFG (a mixture of fructose and glucose at the ratio of 1: 1) at a dose of 0.5 g/kg body weight for another 2 weeks. Subsequently, the mice were sacrificed, and samples were collected for further testing.

The DEX solution was prepared as follows. Initially, 0.5 g of DEX (D829854, Macklin, China) was weighed using an analytical balance and solubilized in 1 mL of dimethyl sulfoxide (DMSO) (D8371, Solarbio, China) to yield a concentrated stock solution of 500 mg/mL. Subsequently, an aliquot of 500 μL from this stock was admixed with 25 mL of PEG400. Upon achieving complete dissolution, the mixture was further diluted with 24.5 mL of saline solution, culminating in the formulation of a working solution with a final concentration of 5 mg/mL.

The MFG solution was prepared by mixing 0.5 g of glucose (G8150, Solarbio, China) and 0.5 g of fructose (F8100, Solarbio, China), which was then dissolved in 20 mL of water.

### 2.3 Mouse Model of MFG‐Promoted Muscle Growth

Twenty 3‐week‐old male C57BL/6 mice were randomly and equally assigned to two groups (*n* = 10). One group was subjected to MFG treatment (0.5 g/kg body weight by gavage) (MFG group); and the other group was gavaged with double distilled water with an equivalent volume to that of the MFG group (CON group). The treatments were commenced at four weeks of age and continued until 8 weeks of age. Subsequently, the mice were sacrificed, and samples were collected for further testing. The total number of mice in each group was *n* = 10 for both the CON and MFG groups.

### 2.4 Mouse Model of BaCl_2_‐Induced Muscle Injury

To induce muscle injury in vivo, the mice were anaesthetized, and the skin was shaved over the tibialis anterior (TA) muscle. Then, 50‐μL 1.2% BaCl_2_ (dissolved in PBS) (B861682, Macklin, China) or PBS was injected into the left or right TA muscle, respectively.

MFG pretreatment was performed 4 days before BaCl_2_ injection and ended 4 or 7 days after injection. Half mice were gavaged with MFG (0.5 g/kg body weight) during this period (MFG group), and the half mice were gavaged with double distilled water (H_2_O group) as the control. The number of mice was *n* = 8 for H_2_O and MFG groups.

### 2.5 Mouse Model of DCA‐Promoted Muscle Growth

Three‐week‐old male C57BL/6 mice were utilized for DCA treatment within their chow diet. Half of the mice were gavaged with DCA at a dose of 10 mg/kg body weight dissolved in saline (DCA group). The other half of the mice were gavaged with saline with an equivalent volume to that of the treatment group as a control (CON group). The treatments were commenced at 4 weeks of age and continued until 8 weeks of age. Subsequently, the mice were sacrificed, and samples were collected for further testing. The total number of mice in each group was *n* = 8 for both the CON and DCA groups.

The DCA solution was prepared by dissolving 10 μg DCA (D2510, Sigma‐Aldrich, USA) in 10 mL of saline.

### 2.6 Mouse Model of AEPR*‐*Promoted Muscle Growth

Male C57BL/6 mice at 3 weeks of age were gavaged with AEPR at a dose of 0.33 g/kg (LAEPR group) and 3.3 g/kg (HAEPR group) body weight of AEPR dissolved in saline. The remaining mice received saline via gavage with an equivalent volume to that of the treatment group as a control (CON group). The treatments were commenced at 4 weeks of age and continued until 11 weeks of age. Subsequently, the mice were sacrificed, and samples were collected for further testing. The total number of mice in each group was *n* = 8 for the CON, LAEPR and HAEPR groups.

PR (Lingkang, Hubei, China) was dried in an oven maintained at 40°C for 3 days. Subsequently, 1‐kg ground PR was prepared for decoction with 5‐L distilled water over a period of 3 h. The solution was subjected to suction filtration, followed by rotary evaporation under reduced pressure until completely dry.

The AEPR solution was prepared by dissolving 6.6‐g AEPR in 20 mL of saline.

### 2.7 C2C12 Cell Culture

Mouse C2C12 cells were provided by ATCC and cultured in DMEM (8119119, Gibco, USA) containing 10% foetal bovine serum and 1% penicillin–streptomycin. When the cells were grown to 80% confluence, the myoblasts were administrated with different concentrations of DCA (0.178, 0.89, 1.78, 8.9, 17.8 μM) (dissolved in DMSO). For TGR5, cAMP and PKA inhibition test, cells were pretreated for 1 h with 100‐μM SBI‐115 (TGR5 inhibitor; S6806, Selleck, China), 100‐μM SQ22536 (cAMP inhibitor; S8283, Selleck, China) or 10‐μM H89 (PKA inhibitor; S1582, Selleck, China) before treatment with DCA.

### 2.8 Luciferase Assay

Two constructs containing the mouse VEGFA or VEGFD promoter (−2000 to +3) bp were generated to PGL3‐basic plasmid. The HEK293T cells were transfected with these plasmids [17] and cells were pretreated for 1 h with 100‐μM SBI‐115, 100‐μM SQ22536 or 10‐μM H89 before treatment with DCA. Luciferase assays were performed with dual luciferase Reporter Assay Kit (DL101‐01, Vazyme, China). Relative luciferase activity (Firefly/Renilla luciferase ratio) was quantified and expressed as the fold change relative to the control group.

### 2.9 Enzyme‐Linked Immunosorbent Assay (ELISA)

Cells were tested with ELISA kits to determine cAMP concentrations. The experiments were performed following the instructions provided in the cAMP Assay Kit (MM‐0544M2, Meimian, China). The samples were measured using a microplate reader.

### 2.10 Histology and Immunofluorescence Staining

The whole‐muscle tissues, including the TA, gastrocnemius (GAS) and quadriceps (Qu), were dissected from the mice and immediately frozen in Tissue‐Tek O.C.T. compound (Sakura, USA). The frozen muscles were then sectioned into 10‐μm thickness slices using a Leica CM1850 cryostat (Leica, Germany).

For H&E staining, the slides containing the muscle sections were first stained in haematoxylin solution for a duration of 20–30 min. Subsequently, the slides were rinsed in running water three times. Following this, the slides were stained in eosin solution for 1–2 min, dehydrated in graded ethanol and xylene and finally covered using neutral gum to prepare for imaging. The determination of the cell area was accomplished by ImageJ.

For immunofluorescence staining, the cross‐sections of the muscle tissue were fixed in 4% paraformaldehyde in PBS for a period of 10 min. After fixation, the samples were incubated in a blocking buffer containing 2% bovine serum albumin in PBS for 1 h at room temperature. Following the blocking step, the samples were incubated with primary antibodies CD31 (1: 200, 28083‐1‐AP, Proteintech), overnight at 4°C.

After the primary antibody incubation, the samples were incubated with Cy3 (GB21403, ServiceBio, China) and DAPI (a fluorescent dye for DNA) (G1407, ServiceBio, China) at room temperature for 1 h. Finally, the samples were covered with a fluorescent mounting medium to prepare for imaging.

### 2.11 Western Blot Analysis

Cells or skeletal muscle samples were lysed with cold RIPA buffer (P0013B, Beyotime Biotechnology, China) containing freshly added phosphatase inhibitor cocktails (P1045, Beyotime Biotechnology, China) on ice for 30 min. Whole cell lysates were centrifuged at 12 000 *g* for 20 min, and the supernatants were transferred into new tubes. Protein concentration of each sample was quantified using a BCA protein assay kit (P0009, Beyotime Biotechnology, China). The same number of proteins (10 μg) were separated by SDS‐PAGE, transferred to PVDF membranes (Milipore, USA), blocked with 5% nonfat milk in TBST buffer (100‐mM NaCl, 10‐mM Tris–HCl, pH 7.5 and 0.1% Tween‐20) for 2 h at room temperature. The following primary antibodies were used at 4°C over night: anti‐MyoD (1:1000, A23881, Abclonal), anti‐MyoG (1:1000, A6664, Abclonal), anti‐MRF4 (1:1000, A15291, Abclonal), anti‐TGR5 (1:1000, DF14067, Affinity), anti‐CREB (1:2000, 12208‐1‐AP, Proteintech), anti‐pCREB (1:1000, 28792‐1‐AP, Proteintech), anti‐Atrogin‐1 (1:2000, 67172‐1‐Ig, Proteintech), anti‐MuRF‐1 (1:2000, 55456‐1‐AP, Proteintech), anti‐GAPDH (1:10 000, 60004‐1‐Ig, Proteintech), HSP90 (1:10 000, A5027, Abclonal). After washing with TBST thrice, a horseradish peroxidase conjugated secondary antibody (1:10 000, A0208, Beyotime) was added and incubated for 1 h at room temperature. Signals were developed using a SuperKine West Pico PLUS Chemiluminescent Substrate kit (Abbkine, Wuhan, China). The intensity of individual bands in western blots was quantified using ImageJ and expressed relative to reference protein signal, as a measure of protein relative abundance in different samples.

### 2.12 Cultivation of *C. aerofaciens*

*C. aerofaciens* was previously isolated in our lab. Plates were incubated for 42 h at 37°C in an anaerobic workstation filled with 80% N_2_, 10% CO_2_ and 10% H_2_. Discrete colonies of *C. aerofaciens* were cultivated in 5 mL of tryptic soy broth.

### 2.13 RNA Extraction, Reverse Transcription and Real‐Time Quantitative PCR (qPCR)

Total RNA was extracted using Trizol reagent (RK30129, ABclonal, China). The integrity and purity of the RNA were determined using a nanodrop spectrophotometer (Thermo Fisher Scientific, USA) and electrophoresis. Complementary DNA was obtained via a two‐step reverse transcription kit (RK20400, ABclonal, China). RNA‐seq was performed by SeqHealth Tech (Wuhan, China).

Using the designed primers, 2 × SYBR Green qPCR Master Mix (ROX2Plus) (A0001‐R2, Abclonal, China) was used in accordance with the stated procedures. The primer sequences used for qPCR are provided in Table S1.

### 2.14 Forced Swimming Test

In brief, the mice were placed in an acrylic plastic pool (50 × 40 × 50 cm) filled with fresh water (25 ± 1°C) to a depth of 30 cm. Exhaustion was defined as loss of coordinated movements and failure to rise to the surface within 7 s, and the swimming time was recorded as previously described [18].

### 2.15 Grip Strength Test

A grip strength meter (Shence, Shenzhen, China) was used to measure the grip strength as previously described [19]. Briefly, the mice were restrained properly by holding the scruff and base of the tail and allowed to grasp the angled grid. Mice were then gently pulled along the length of the sensor grid until the grip was released. The displayed value in the ‘N’ force was recorded with each animal tested for three times to obtain an average value of the grip strength.

### 2.16 Metabolomic Profiling

Untargeted metabolomics was conducted by BioDeep (Suzhou, China). The experimental procedure included metabolite extraction and LC–MS/MS detection. Briefly, 25 mg of tissue was weighed and extracted by directly adding 800 μL of precooled extraction reagent (methanol:acetonitrile:water [2: 2:1, v/v/v]). Internal standard mixture was added for quality control (QC) of sample preparation. The samples were homogenized for 5 min using TissueLyser (JXFSTPRP, China), followed by 10 min of sonication and an hour of incubation at −20°C. The supernatant from the centrifugation of the samples was then transferred for vacuum freeze drying after 15 min at 25 000 rpm and 4°C. The metabolites were re‐suspended in 600 μL of 10% methanol and sonicated for 10 min at 4°C. After centrifugation for 15 min at 25 000 rpm, the supernatants were transferred to autosampler vials for LC–MS analysis. To evaluate the consistency of the entire LC–MS analysis, a QC sample was created by pooling the same volume of each sample. We employed a tandem Q Exactive high‐resolution mass spectrometer from Thermo Fisher.

### 2.17 Determination of 16S rRNA Gene Sequence and Bacterial Abundance

The 16S rRNA gene sequencing was performed by Personalbio (Shanghai, China). The PCR reaction system was configured using 30 ng of qualified genomic DNA samples and the corresponding fusion primers (338F: ACTCCTACGGGAGGCAGCAG, 806R: GGACTACHVGGGTWTCTAAT), and the corresponding PCR reaction parameters were set for PCR amplification. The PCR amplification products were purified, and library construction was completed. The libraries were assessed for fragment range and concentration using an Agilent 2100 Bioanalyzer. The libraries that passed the test were sequenced by selecting the appropriate platform (HiSeq/MiSeq) according to the insert size. The downstream data were filtered to remove low‐quality reads, and the remaining high‐quality clean data were used for further analysis. The reads were stitched into tags based on the overlapping relationship between reads. The tags were then clustered into operational taxonomic units (OTUs) at a given similarity, and the OTUs were compared with the database for species annotation. Species annotation was performed based on the OTUs and the species annotation results for species complexity analysis and species difference analysis between groups.

### 2.18 DCA Measurement in Bacterial Culture

The culture medium used for in vitro DCA synthesis comprised trypsin (10 g/L), beef extract (10 g/L), yeast extract (3 g/L), glucose (5 g/L), NaCl (5 g/L), soluble starch (1 g/L), L‐cysteine HCL (0.5 g/L), sodium acetate (3 g/L), resazurin (4 mL/L) and chenodeoxycholic acid (5 g/L). DCA was measured by bile acid profiles as previously described [20].

### 2.19 Data Analysis

Statistical analysis was performed using Prism 8 software (GraphPad Software, San Diego, CA, USA). Student's *t*‐test was used to analyse the statistical significance between two groups. Data are expressed as means ± standard deviation (SD). *p < 0.05, **p < 0.01, ***p < 0.001 and ns represents not significant.

## 3 Results

### 3.1 AEPR Promotes the Growth and Force of Skeletal Muscle

**FIGURE 1:**
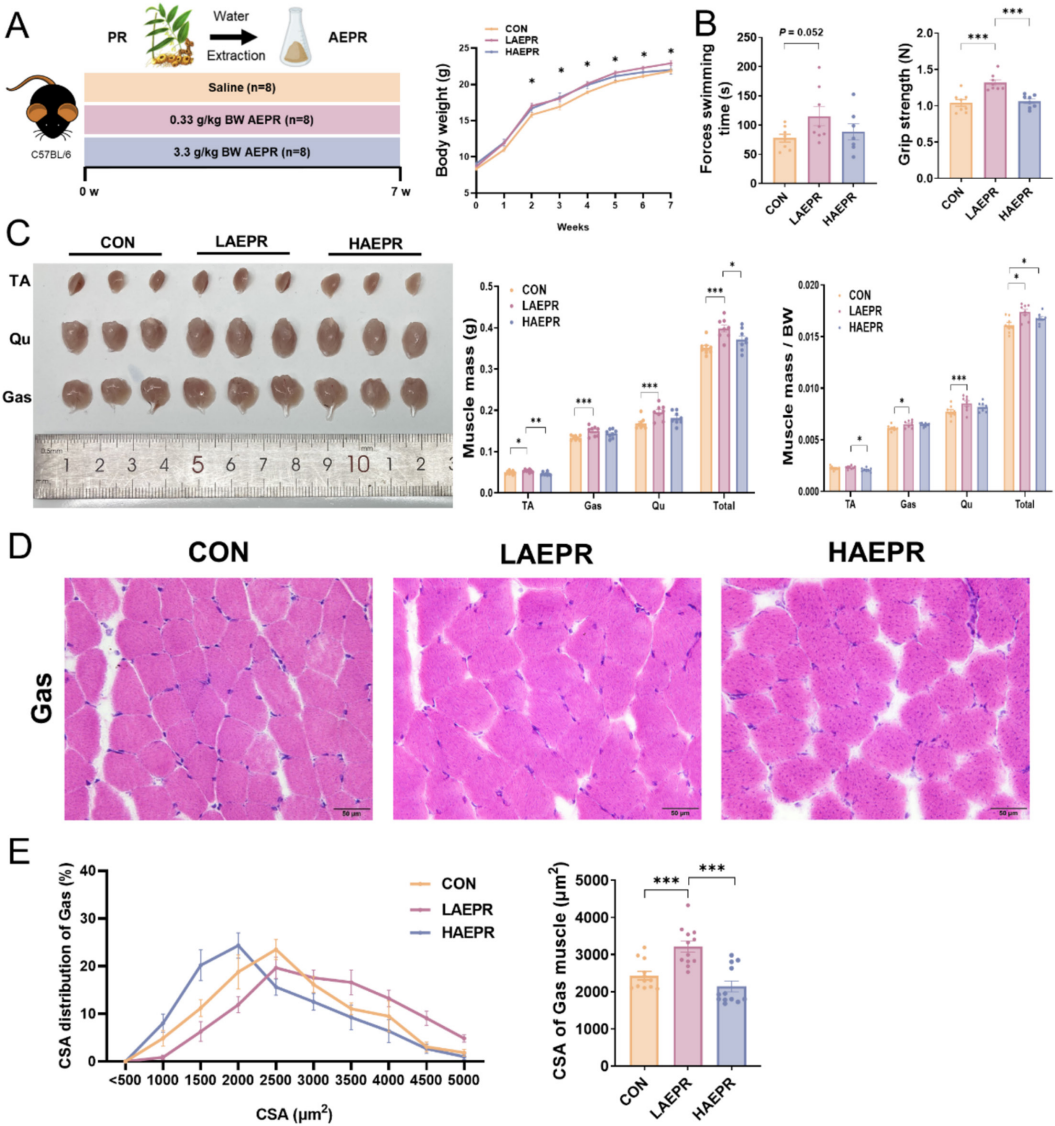
AEPR promotes growth and force of skeletal muscle. (A) Study design with different concentrations of AEPR, *n* = 8 biological replicates in each group and body weight curve under different treatments; (B) forced swimming times of mice and grip strength of mice; (C) representative images of TA, Qu and GAS muscles in the CON, LAEPR and HAEPR mice and muscle mass; (D and E) representative H&E staining of myofibre cross‐section of GAS and cross‐sectional area (CSA) distribution and average CSA of muscle fibre. Data information: *t* test was used in this figure where error bars represent SD; and *p < 0.05; **p < 0.01; ***p < 0.001.

PR is also known as HuangJing and its rhizome is often used as a medicine. It is one of the most famous medicine food homology herbals. PR has been shown to alleviate myocardial ischemia, particularly with its anti‐fatigue effects, indicating its potential in treating muscular diseases [21, 22]. Thus, we first investigated whether PR has certain effects on muscle development. Three‐week mice were gavaged with AEPR at lower (LAEPR) and higher (HAEPR) concentrations (Figure 1A). The results showed that LAEPR treatment increased the body weight of mice (Figure 1A), but had no apparent effect on their food intake (Figure S1A). To verify whether AEPR influences the performance of skeletal muscle, we conducted grip strength and forced swimming test. LAEPR elevated the grip strength and swimming time, but HAEPR showed no such effect (Figure 1B) and increased the liver mass of mice (Figure S1B). We then isolated various muscle tissues, including TA, GAS and Qu from the CON, LAEPR and HAEPR groups. Consistent with the increased body weight in LAEPR group, LAEPR also significantly increased the weights and ratios of TA, GAS and Qu (Figure 1C). H&E staining further showed that the fibres in GAS muscle from the LAEPR group were larger than those of CON group (Figure 1D,E). These results suggested that gavage with AEPR can promote the growth and force of mouse skeletal muscle.

### 3.2 MFG Promotes Muscle Development and Regeneration

**FIGURE 2:**
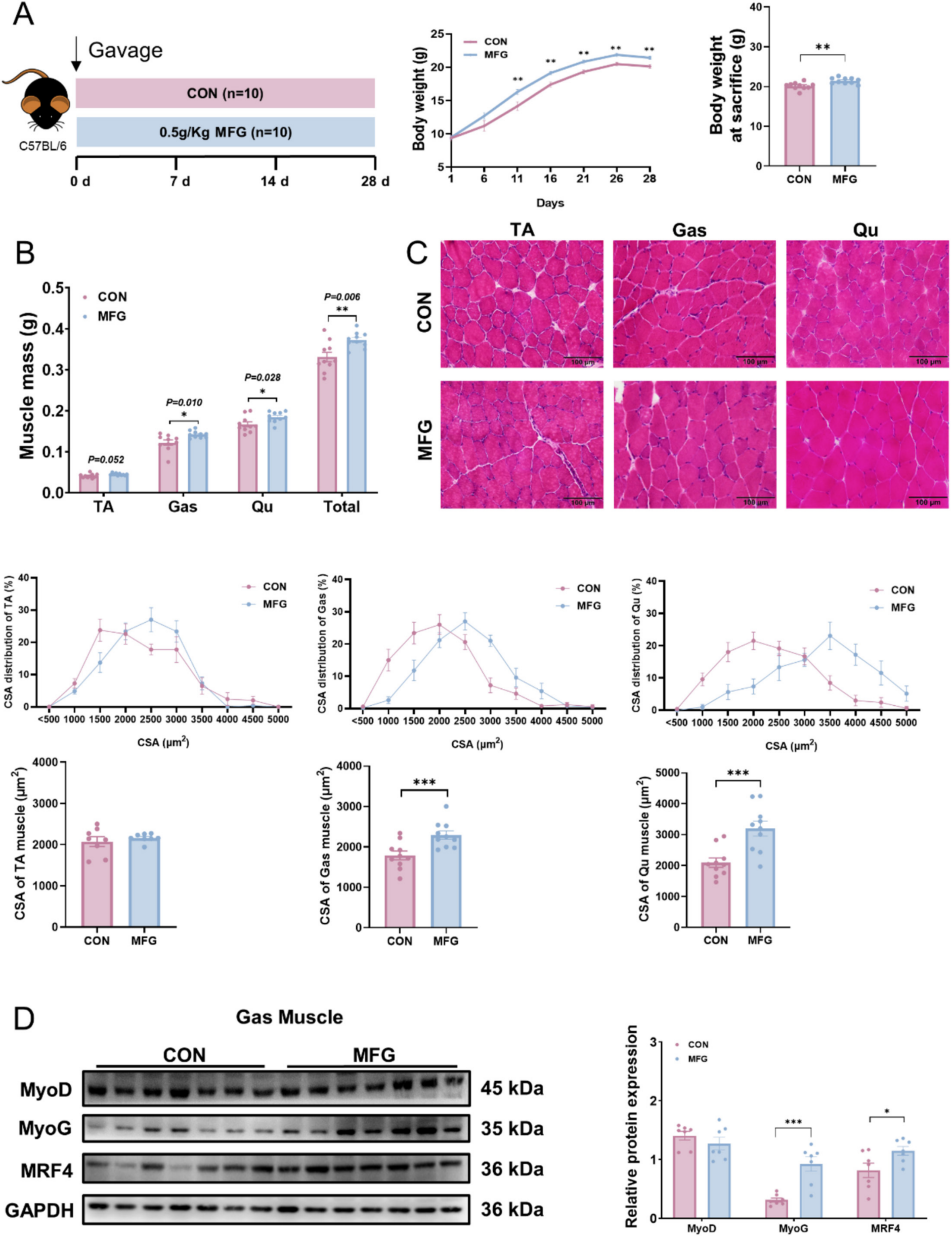
MFG promotes muscle development. (A) Study design of MFG treatment, *n* = 10 biological replicates in each group and body weight curve under different treatments; (B) TA, GAS and Qu muscle mass; (C) representative H&E staining of myofibre cross section of TA, GAS and Qu and the CSA distribution and average CSA of muscle fibre; (D) western blot and quantification of MyoD, MyoG and MRF4; Data information: *t* test was used in this figure where error bars represent SD; and *p < 0.05; **p < 0.01; ***p < 0.001.

PR is composed of various monosaccharides, including fructose, glucose and some other monosaccharides [21, 22]. We next determined whether gavage of MFG also exhibited beneficial effect on muscle growth and force. We gavaged 3‐week‐old C57BL/6 male mice with MFG or water (control). MFG treatment increased the body weight and muscle mass of mice (Figure 2A,B), but did not change their food intake (Figure S1C). H&E staining showed that MFG‐treated mice had larger muscle fibre area than those of CON group (Figure 2C). In addition, the western blotting results revealed that MFG treatment significantly increased the protein levels of MyoG and MRF4 in GAS (Figure 2D). In addition, the results demonstrated that MFG treatment had no effect on liver weight, serum alanine aminotransferase (ALT), serum aspartate aminotransferase (AST), hepatic or serum triglycerides (TG) and total cholesterol (TC), serum uric acid (UA) and myocardial indicators myohaemoglobin (Mb) and cardiac troponin I (cTnI) (Figure S1). Our data demonstrate that MFG, which can represent the active components in AEPR, promotes muscle development of mice.

Considering the beneficial effects of MFG and the elevated level of MyoG, we explored whether MFG gavage also promotes muscle regeneration. To this end, we established a BaCl_2_‐induced mouse muscle injury model to dissect the effects of MFG on muscle regeneration (Figure S2A,D). As a result, MFG supplementation attenuated the decrease in muscle weight induced by BaCl_2_ injection at 4 and 7 days after muscle injury (Figure S2B,E). We then conducted H&E staining to further examine the repairing effect of MFG on skeletal muscle injury. The results showed that MFG supplementation did not affect the percentage of necroptotic area at day 4, but increased the area of the regenerated muscle fibres as indicated by central‐nuclei at Day 7 after BaCl_2_ injection (Figure S2C,F), indicating enhanced muscle repairing. Taken together, MFG could promote muscle development and muscle regeneration of the mice.

### 3.3 MFG Alleviates DEX‐Induced Muscle Growth Inhibition

**FIGURE 3:**
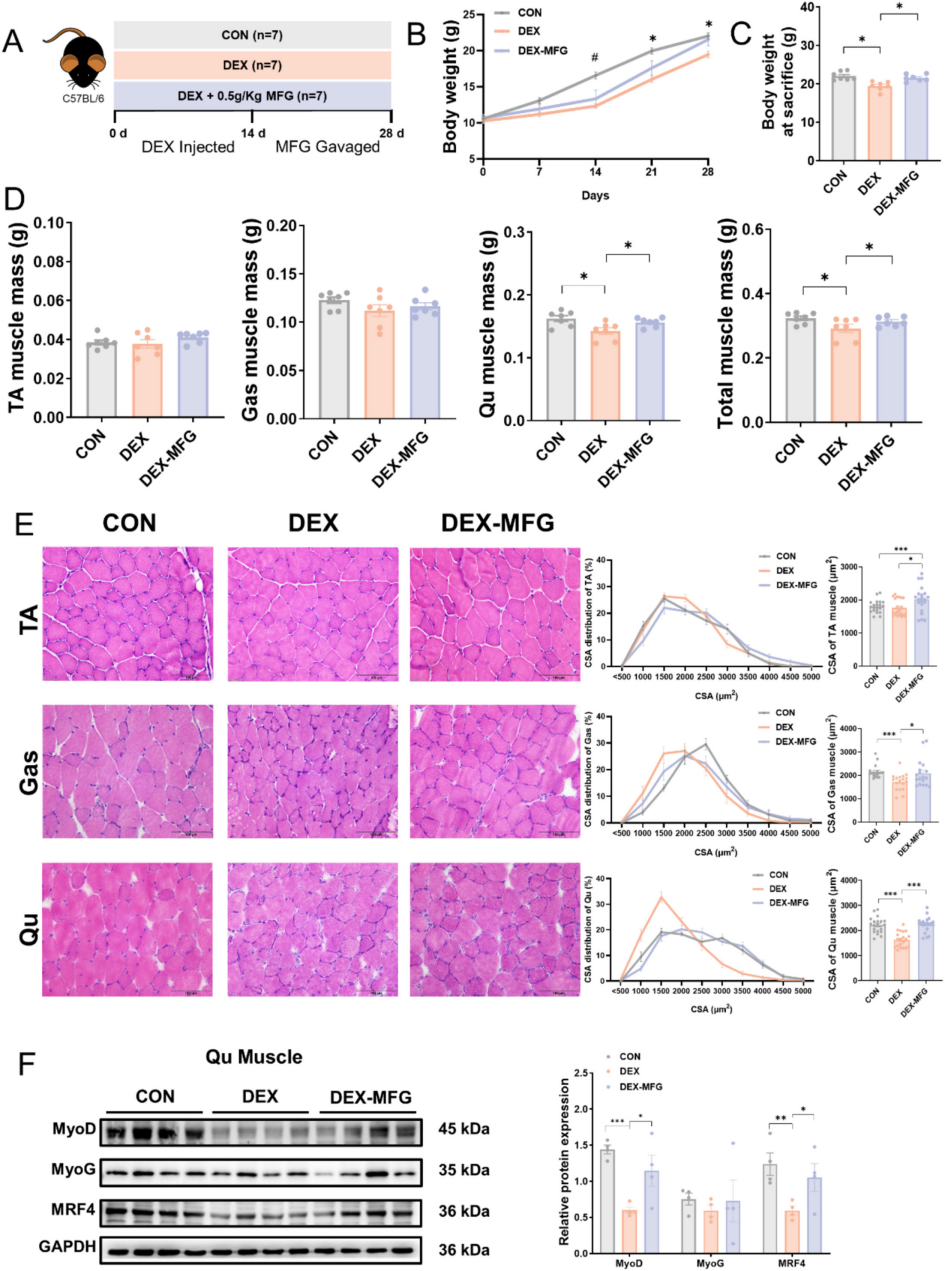
MFG alleviates glucocorticoid‐induced muscle growth inhibition. (A) Study design of the DEX‐induced muscle growth inhibition model, *n* = 7 biological replicates in each group; (B) body weight curve under different treatments; (C) body weight; (D) TA, GAS, Qu and total muscle mass; (E) representative H&E staining of myofibre cross section of TA, GAS and Qu and CSA distribution and average CSA of muscle fibre; (F) western blot and quantification of MyoD, MyoG and MRF4. Data information: *t* test was used in this figure where error bars represent SD; and *p < 0.05; **p < 0.01; ***p < 0.001.

DEX is a synthetic glucocorticoid and can increase the risk of muscle growth inhibition due to its catabolic effects on protein metabolism, including enhanced proteolysis (protein breakdown) and suppressed protein synthesis [3]. Thus, we applied DEX‐induced muscle growth inhibition model to explore the protective effects of MFG against muscle growth inhibition (Figure 3A). The DEX group exhibited a significant decrease in food intake compared with the CON group, while the DEX‐MFG group showed no significant change in food intake (Figure S3A). After 14 days of gavage, MFG significantly increased the body weight of the mice compared with those of DEX group (Figure 3B,C). We then evaluated the weights of various muscles, and the results showed that DEX decreased the mass of Qu, whereas MFG supplementation restored the mass of Qu in DEX‐treated mice (Figure 3D). H&E staining confirmed that the significantly reduced muscle fibre areas in DEX‐treated mice were restored by MFG supplementation (Figure 3E). Western blotting results demonstrated that MFG alleviated the decline in protein levels of MyoD and MRF4 caused by DEX in the TA muscle (Figure 3F). We also examined the effects of MFG on the liver, adipose tissue and intestine. The results showed that compared with DEX treatment, MFG had no significant impact on adipose tissue weight or the length of the ileum and colon (Figure S3B–D). When assessing whether fructose or glucose contributes to the effect of MFG, we found that supplement of fructose alone had the same effect as MFG to increase the body weight, muscle mass and fibre area of mice (Figure S4A–D), but exhibited no significant effect on muscle atrophy‐related proteins and myogenic regulatory factors (Figure S4E,F). Moreover, the fructose, glucose or MFG treatment did not cause fatty liver in mice (Figures S4G and S6F–H), and had no effects on serum UA, Mb and cTnI (Figure S6I,J). These results demonstrated that MFG administration relieves DEX‐induced skeletal muscle growth inhibition in mice.

### 3.4 MFG Promotes Muscle Angiogenesis via VEGFs to Alleviate DEX‐Induced Muscle Growth Inhibition

**FIGURE 4:**
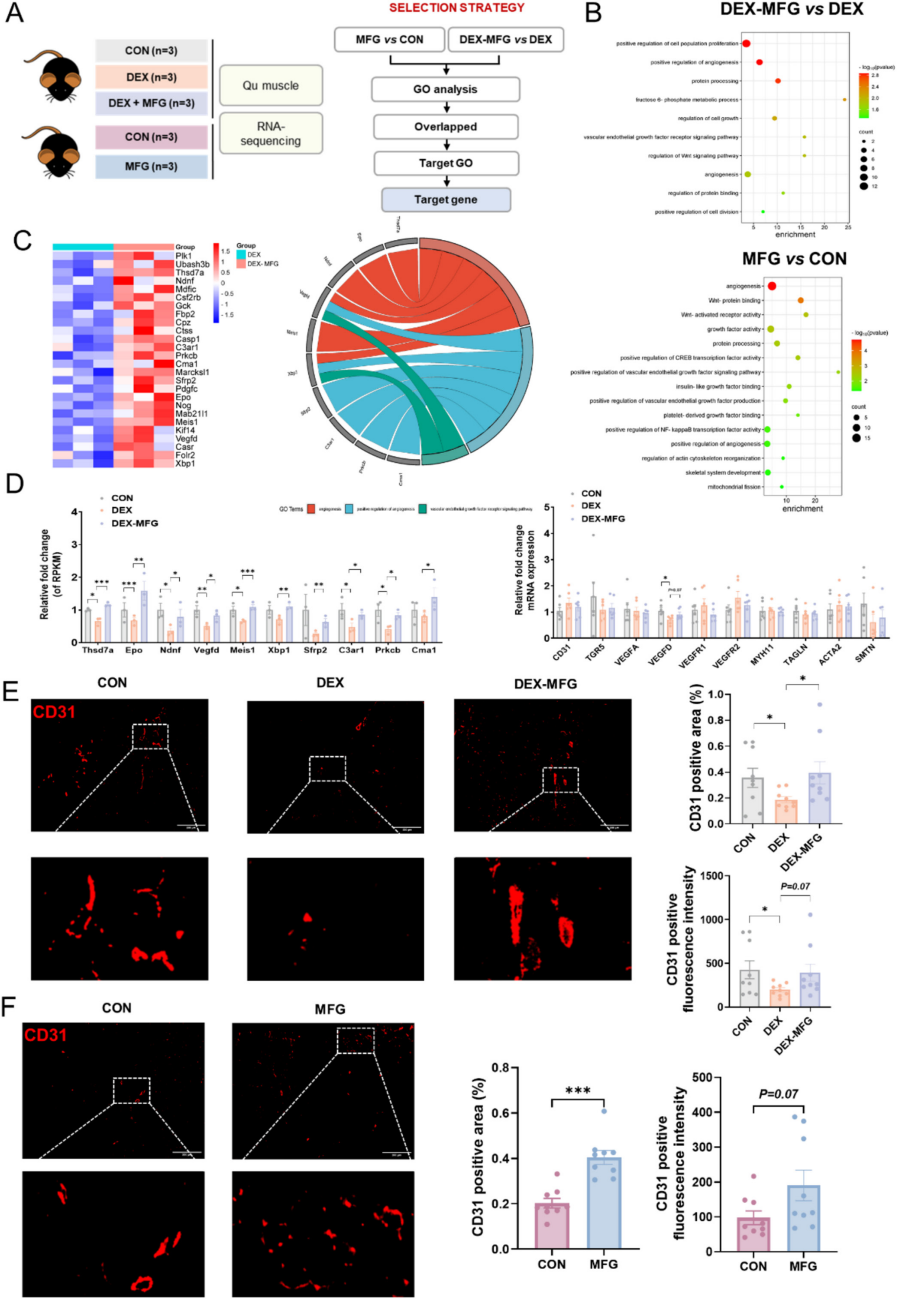
MFG promotes muscle angiogenesis via VEGFs to alleviate glucocorticoid‐induced muscle growth inhibition. (A) Groups of mRNA sequencing, *n* = 3 biological replicates in each group and work flow of selection strategy of mRNA sequencing; (B) GO pathway enrichment analyses of the upregulated DEGs in MFG versus CON and DEX‐MFG versus DEX, the dot size represents the number of DEGs and the dot colour represents the corresponding *p* value; (C) heatmap of representative DEGs in DEX‐MFG versus DEX and representative significantly upregulated GO terms with genes in DEX‐MFG versus DEX; (D) RPKM levels of representative genes in the control (CON), DEX and DEX‐MFG and mRNA levels of angiogenesis and contractility genes in DEX‐MFG group; (E and F) representative images and quantification of CD31 immunofluorescence staining in Qu muscle of DEX‐MFG group and MFG group. Data information: *t* test was used in this figure where error bars represent SD; and *p < 0.05; **p < 0.01; ***p < 0.001.

To further dissect the signalling pathways related to the MFG‐mediated beneficial effects, we performed RNA sequencing to discover the differentially expressed genes (DEGs) in Qu muscles from the DEX‐MFG and MFG groups (Figure 4A). In particular, gene ontology (GO) pathway enrichment analysis showed that the upregulated DEGs were significantly enriched in angiogenesis‐related pathways (Figure 4B,C). Moreover, MFG supplementation upregulated angiogenesis‐related genes in the DEX‐induced muscle growth inhibition model, especially *VEGFD* (Figure 4D). qPCR validation confirmed that the mRNA expression of *VEGFD* was significantly suppressed by DEX, while the suppression was ameliorated by MFG supplementation (Figure 4D). Immunofluorescence of CD31, a vascular endothelial marker, confirmed that both the MFG and DEX‐MFG groups showed increases in the expression of CD31 (Figure 4E,F). We further determined whether gavage of AEPR could also promote the angiogenesis of skeletal muscle. The qPCR results showed that LAEPR treatment upregulated the mRNA expression levels of *VEGFA* and *VEGFD* in Qu muscle, while LAEPR treatment or fructose treatment resulted in intensified immunofluorescence signal of CD31 in Qu muscle, suggesting that LAEPR and fructose could also enhance the angiogenesis ability of skeletal muscle in mice (Figure S5A–D). These results indicated that MFG upregulates VEGFs and enhances angiogenesis in skeletal muscles to promote muscle growth and alleviate glucocorticoid‐induced muscle growth inhibition.

### 3.5 MFG Promotes Muscle Angiogenesis Through Increasing Intestine‐Derived DCA Production

**FIGURE 5:**
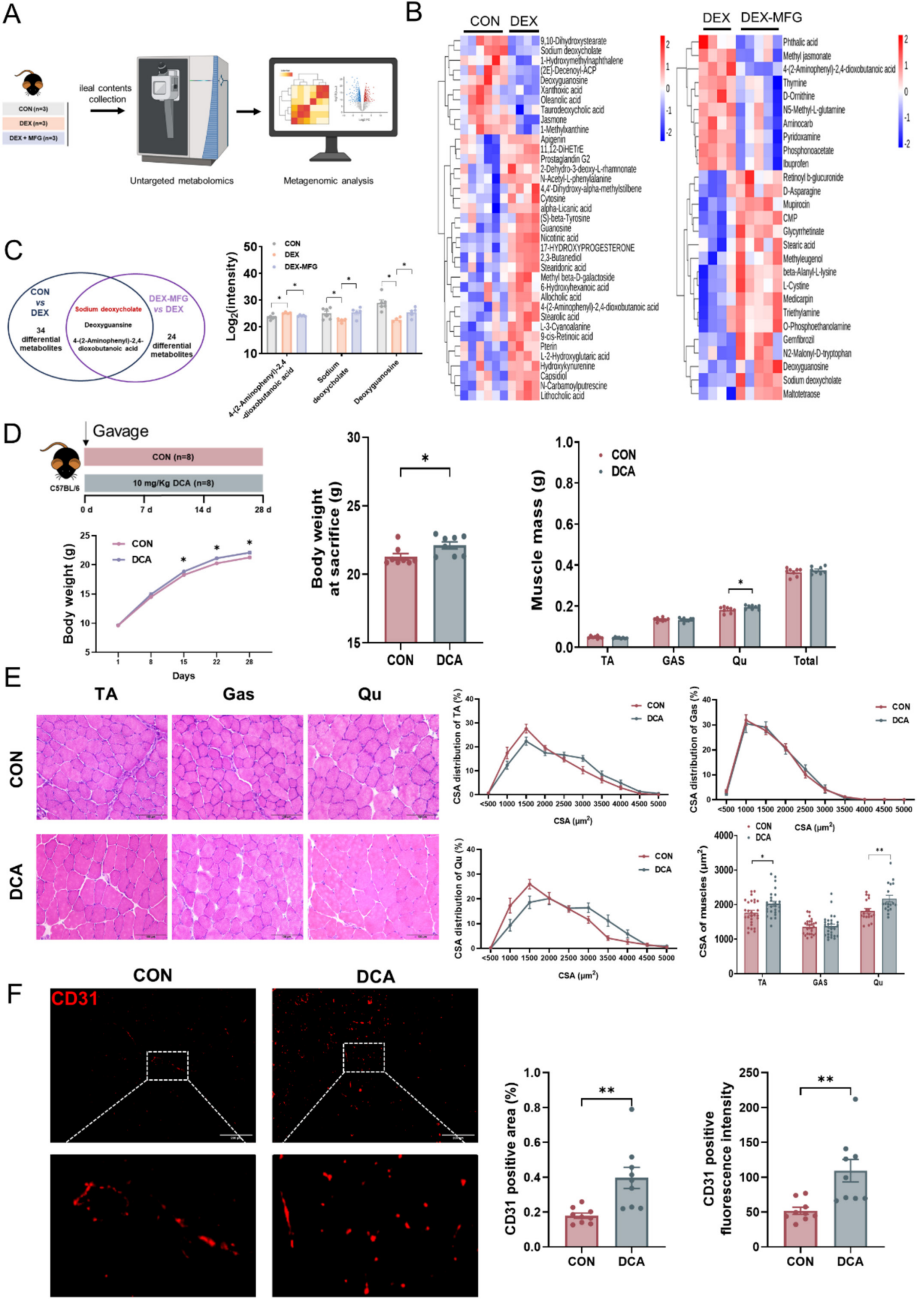
MFG promotes muscle angiogenesis through increasing intestine‐derived DCA production. (A) Groups of non‐targeted metabonomic sequencing, *n* = 3 biological replicates in each group; (B) heatmaps of representative metabolites in ileal contents in mice of the DEX versus control (CON) groups, and DEX‐MFG versus DEX groups; (C) Venn plot of common and distinct differential metabolites of DEX versus CON and DEX‐MFG versus DEX and relative enrichment of common differential metabolites measured by LC–MS/MS; (D) body weight curve under different treatments, *n* = 8 biological replicates in each group and muscle mass; (E) representative H&E staining of myofibre cross section of TA, GAS and Qu and CSA distribution and average CSA of muscle fibre; (F) representative images and quantification of CD31 immunofluorescence staining in Qu muscle of DCA group. Data information: *t* test was used in this figure where error bars represent SD; and *p < 0.05; **p < 0.01; ***p < 0.001.

Previous research has suggested that intestine clears approximately 90% of low doses of fructose, resulting in the entry of only a small amount of fructose into circulation, while significant levels of glucose, lactate and glycerate derived from fructose are detected in the portal blood [16]. Furthermore, previous studies have reported that a diet containing both fructose and glucose can lead to changes in gut microbiota and metabolites [23]. In this study, untargeted metabolomics revealed that DEX treatment significantly reduced the abundance of sodium deoxycholate and deoxyguanosine and increased that of dioxobutanoic acid, while MFG supplementation notably increased the abundance of sodium deoxycholate and deoxyguanosine and attenuated the increase in dioxobutanoic acid (Figure 5A–C). DCA is a secondary bile acid, and DCA in bile exists in the form of sodium deoxycholate. Because DCA is related to nutrient metabolism, we investigated whether DCA is responsible for the beneficial effects of MFG in promoting muscle development of mice. To this end, 3‐week‐old C57BL/6 male mice were gavaged with DCA. Consistent with the effects of MFG, DCA treatment increased the body weight and muscle mass of mice (Figure 5D), but did not change their food intake (Figure S6A). DCA treatment increased the fat mass of mice (Figure S6B). H&E staining also showed that muscle fibre area in TA and Qu from the DCA group were larger than those from the CON group (Figure 5E). Consistently, DCA significantly upregulated the mRNA expression of *VEGFs* (Figure S6C), and increased the expression of CD31 in muscle (Figure 5F). Collectively, MFG gavage promotes the growth and angiogenesis of mouse skeletal muscle by increasing the abundance of DCA in the intestine.

### 3.6 DCA Is Produced by *C. aerofaciens* in Response to MFG Supplementation

**FIGURE 6:**
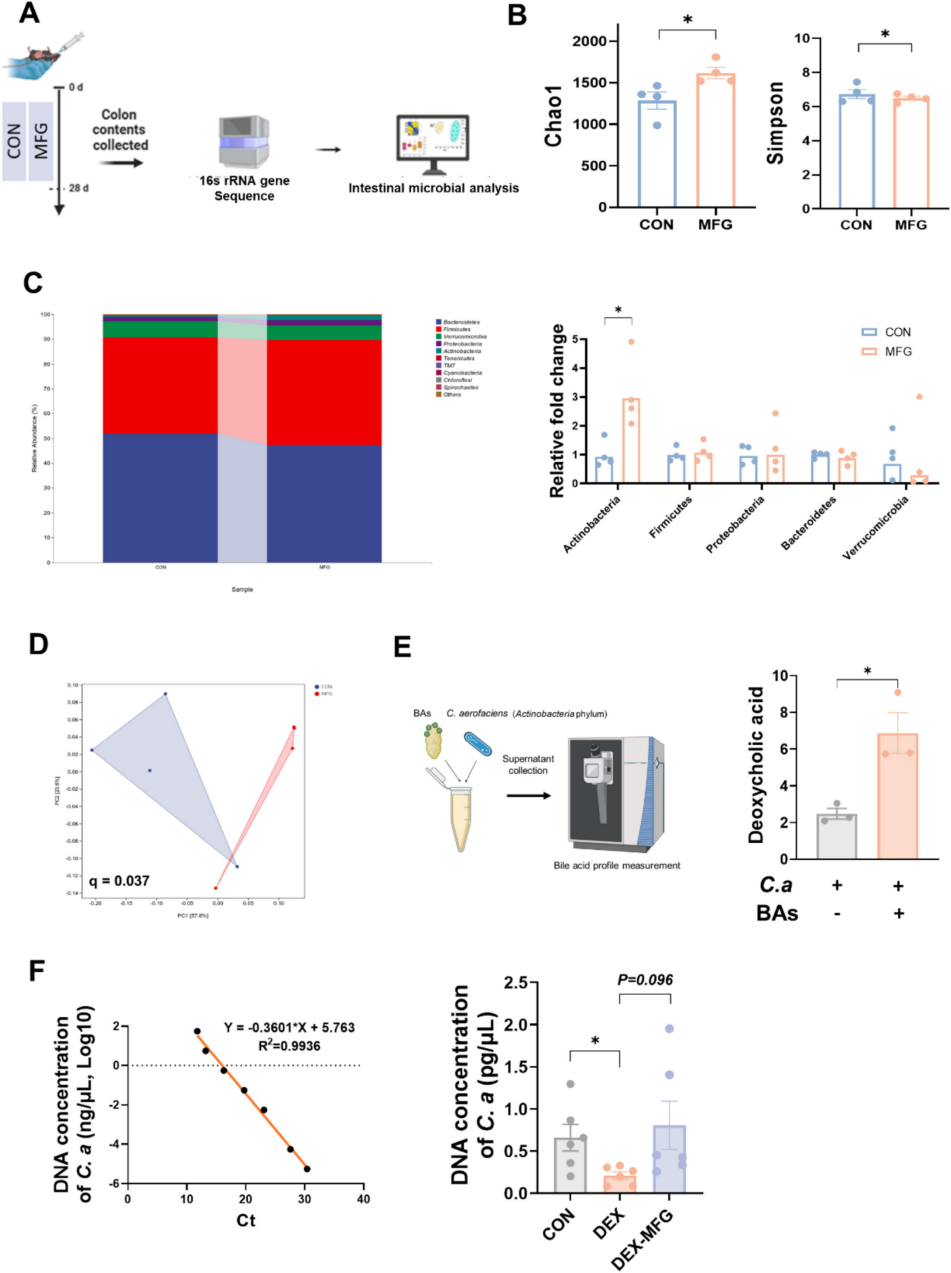
DCA is produced by *C. aerofaciens* in response to MFG supplementation. (A) Groups of 16S rRNA gene high‐throughput sequencing, *n* = 4 biological replicates in each group; (B) α‐diversity analysis of gut microbes reflected by Chao1 and Simpson indices; (C) gut microbiota abundance at the phylum level and comparison of phyla in the CON and MFG groups; (D) principal component analysis plot of the colonic contents in the CON and MFG groups; (E) bile acid profile measurement, *n* = 3 biological replicates in each group and DCA synthesis in vitro; (F) standard curve of *C. aerofaciens* and DNA concentrations of *C. aerofaciens* in DEX‐MFG group, *n* = 6 biological replicates in each group; *t* test was used in this figure where error bars represent SD; and *p < 0.05.

Secondary bile acid is produced from primary bile acid by gut microbiota. Considering the change in DCA abundance, we determined whether there was a change in gut microbiota after MFG treatment, especially DCA‐producing bacteria, by 16S rRNA gene sequencing (Figure 6A). The results showed that the alpha diversity of the intestinal microbiota, as represented by Chao1 and Simpson index, increased in MFG group compared with that of the CON group (Figure 6B). Principal coordinates analysis (PCoA) revealed significant variations in intestinal microbiota community between the two groups (Figure 6D). At the phylum level, MFG treatment significantly increased the abundance of *Actinobacteria* (Figure 6C). *C. aerofaciens* is a highly abundant member of the *Actinobacteria* phylum, which is positively correlated with the production of secondary bile acids [24]. We hypothesized that *C. aerofaciens* may be a responsible for the increased DCA production after MFG treatment. In line with our hypothesis, *C. aerofaciens* elevated the level of DCA upon the addition of chenodeoxycholic acid (a primary bile acid) in the culture supernatant (Figure 6E). Next, we used the prepared standard curve to detect the abundance of the bacterial DNA in the colonic chyme. As a result, DEX treatment significantly reduced the abundance of *C. aerofaciens* , while MFG supplementation restored it to a normal level (Figure 6F). Furthermore, the results showed that DEX treatment had no influence on hepatic total bile acids, cholic acid (CA) or bile acid synthesis‐related genes *CYP7A1*/*CYP8B1* (Figure S6D,E), suggesting that DEX may not influence CA production (CA is the precursor of DCA) by regulating hepatic bile acid synthesis pathways. These results indicated that MFG supplementation could promote the production of DCA by *C. aerofaciens* .

### 3.7 TGR5/cAMP/PKA/pCREB Pathway Accounts for the Upregulation of VEGFs in Muscle Angiogenesis

**FIGURE 7:**
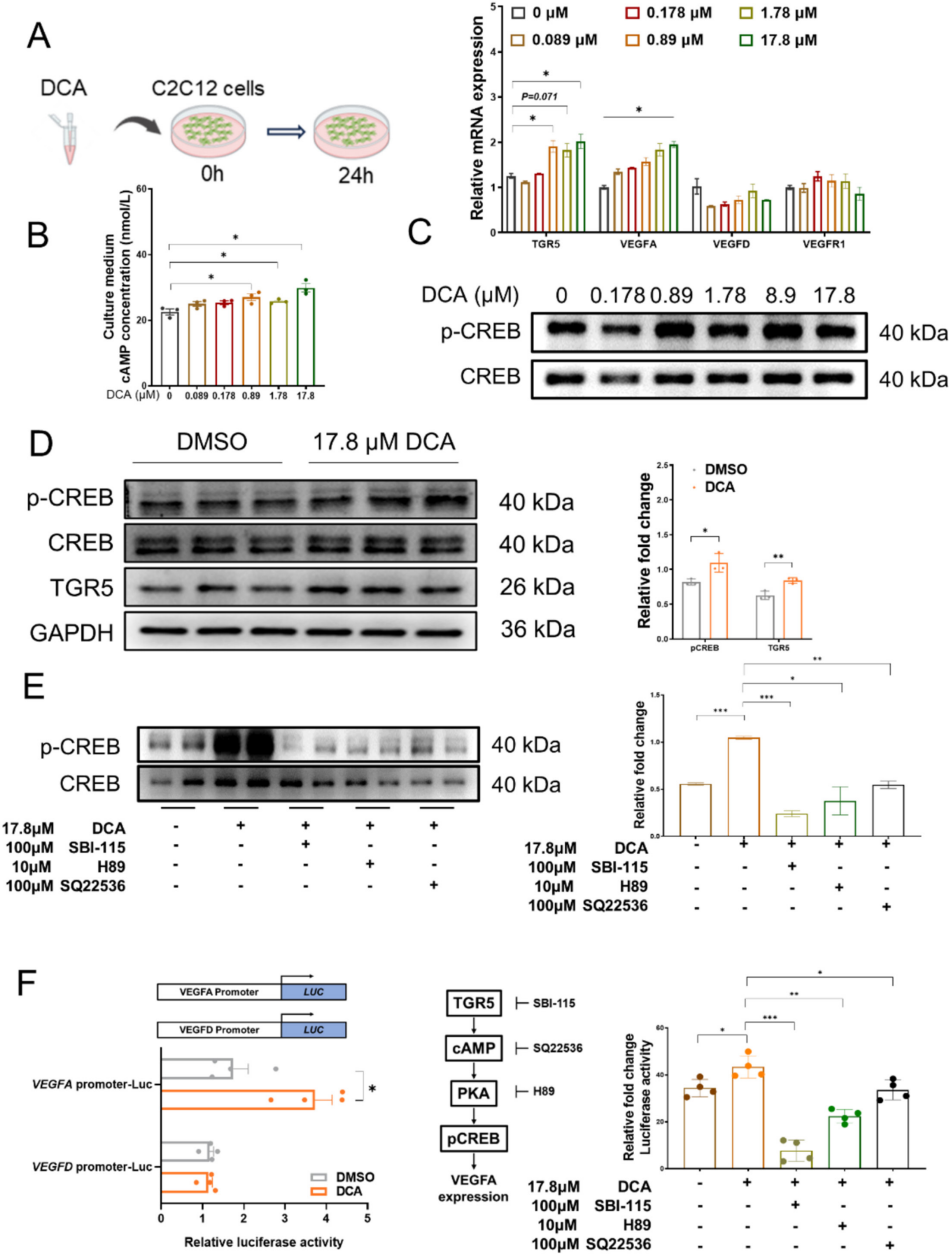
TGR5/cAMP/PKA/pCREB pathway accounts for the upregulation of VEGFs in muscle angiogenesis. (A) Study design of the C2C12 cells with DCA treatment, *n* = 3 biological replicates in each group and mRNA levels of genes under treatments with different concentrations of DCA; (B) cAMP concentration of culture medium; (C) Western blot of p‐CREB under treatments with different concentrations of DCA; (D) western blot and quantification of p‐CREB and TGR5 in DCA treatment groups; (E) western blot and quantification of p‐CREB under treatments with DCA and inhibitors; (F) luciferase activity with DCA treatment, *n* = 4 biological replicates in each group and luciferase activity under DCA and inhibitor treatments. Data information: *t* test was used in this figure where error bars represent SD; and *p < 0.05; **p < 0.01; ***p < 0.001.

**FIGURE 8:**
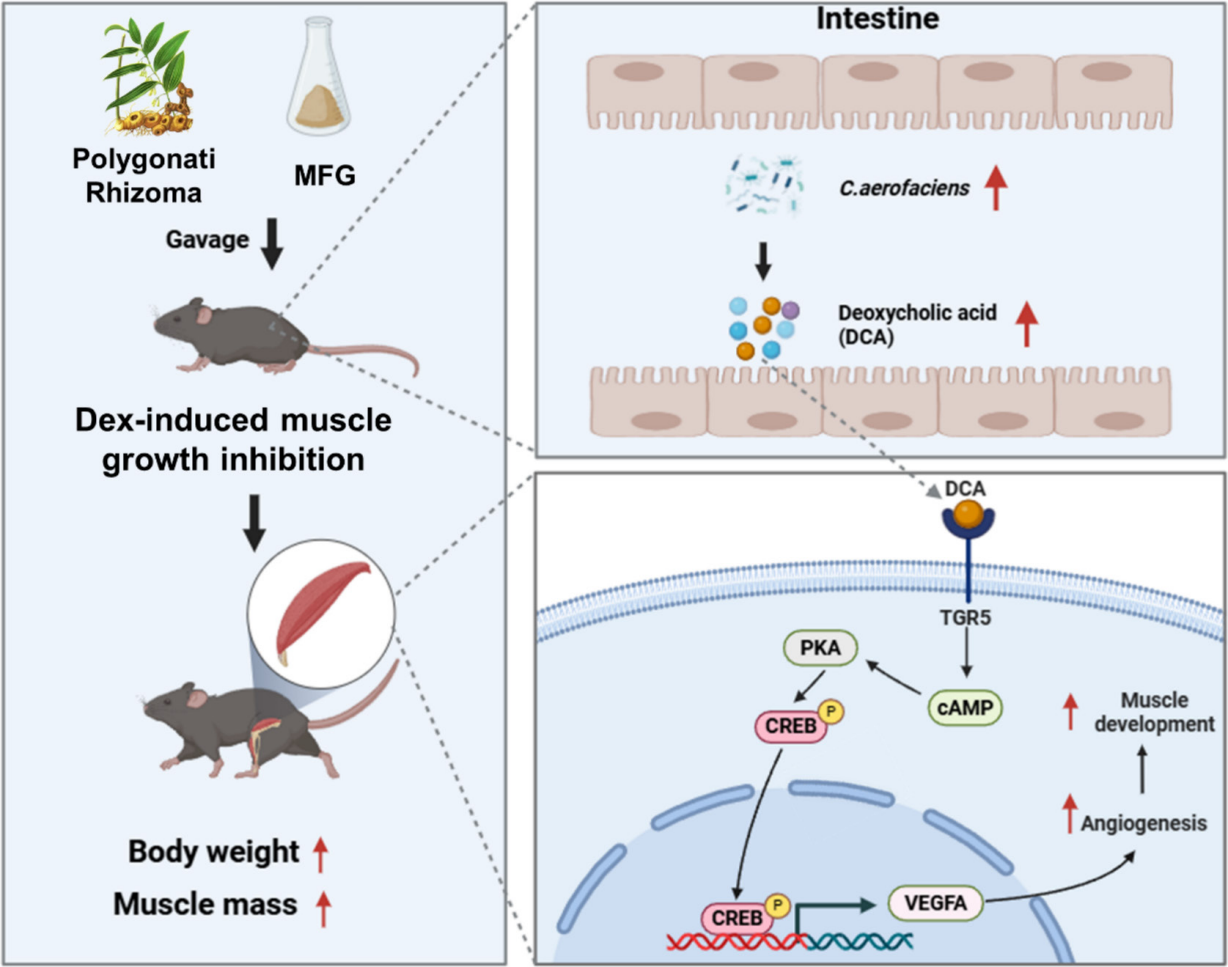
Mechanism diagram for MFG to ameliorate glucocorticoid‐induced skeletal muscle growth inhibition in mice.

We then attempted to determine how DCA produced by gut microbiota elevates the VEGFs level. TGR5 is a membrane receptor for bile acids in muscle, which sequentially activates adenylate cyclase and results in an increase in cyclic AMP (cAMP). We observed that DCA treatment increased the mRNA levels of *VEGFA* and *TGR5* in C2C12 myoblasts (Figure 7A), as well as the cAMP level (Figure 7B). To elucidate the signalling pathway that links TGR5 to *VEGFA* expression, we examined the CREB family, which is known for its role in VEGFA transcriptional regulation [25, 26]. We found that DCA increased the expression of p‐CREB/CREB (Figure 7C). Particularly, 17.8‐μM DCA increased the expression of both TGR5 and p‐CREB (Figure 7D). In contrast to DCA, the TGR5 inhibitor SBI‐115, the PKA inhibitor H89 and the cAMP inhibitor SQ22536 markedly decreased the expression of p‐CREB/CREB in C2C12 (Figure 7E). To further elucidate whether DCA modulates the transcriptional activity of pCREB, we cloned the promoter region of *VEGFA* or *VEGFD* into PGL3‐basic and performed dual‐luciferase reporter assay. The results revealed that the transcriptional activity of the *VEGFA* promoter was increased by DCA treatment, but decreased by SBI‐115, SQ22536 or H89 (Figure 7F). Taken together, DCA increases the *VEGFA* level via the TGR5/cAMP/PKA/pCREB axis Figure 8.

## 4 Discussion

Our experimental results shed light on the potential protective effects of PR and MFG against muscle growth inhibition, as well as their role in promoting muscle angiogenesis. AEPR and MFG treatment modified the gut microbiota, increasing the abundance of *C. aerofaciens* , which further promoted muscle development by increasing the expression of angiogenesis‐related genes such as VEGFs via the DCA‐myocyte TGR5/cAMP/PKA/pCREB axis.

Several studies have associated excessive consumption of HFCS with obesity and metabolic diseases [23, 27]. However, a recent report suggests that dietary fructose at a lower concentration (25% HFCS in tap water) enhances intestinal villus length and improves nutrient absorption in mouse models [15], indicating the possibility that fructose at low concentrations may promote nutrient absorption without causing metabolic diseases. It has been reported that low doses of fructose (approximately 90% clearance by the intestine) are well‐tolerated, while high doses (> 1 g/kg) will overwhelm intestinal absorption and clearance ability, leading to spillage of fructose into the liver [16]. Excessive fructose consumption is known to contribute to nonalcoholic fatty liver disease (NAFLD) [28]. NAFLD comprises various disorders ranging from simple steatosis to nonalcoholic steatohepatitis, which can progress to fibrosis, cirrhosis and liver cancer [29]. In this study, we assessed the indicators of fatty liver in mice. The results suggested that the fructose doses used in this study (lower than 1 g/kg) are safe for mouse liver, which is in line with previous finding that low doses of fructose can be effectively cleared by the intestine without causing liver metabolic diseases such as NAFLD [16]. In addition, fructose is a major mediator of cardiovascular disease risk starting at paediatric age and is known to increase blood uric acid that can cause gout [30]. Our study focused on acute muscle‐protective effects rather than long‐term outcomes. Our future work will evaluate the age‐dependent responses and long‐term safety. To balance potential benefits and risks, subsequent research should compare the metabolic effects of short‐term therapeutic fructose use versus chronic dietary exposure, explore synergies with uric acid‐lowering agents (such as allopurinol) to mitigate hyperuricemia risks, and investigate tissue‐specific fructose metabolism to decouple muscle‐protective effects from systemic harm [31].

The satellite cell niche and angiogenesis are intimately connected in the context of muscle regeneration [32, 33]. Satellite cells are the primary stem cells responsible for muscle repair and reside in a specialized microenvironment that is crucial for their function and maintenance [34]. This niche is influenced by various factors such as blood vessels. Angiogenesis, a process of forming new blood vessels, plays a vital role in supplying oxygen and nutrients to the regenerating tissue [32], as well as facilitates the removal of waste products [35]. The proximity of satellite cells to capillaries is crucial, as endothelial cells (ECs) can secrete growth factors and cytokines to directly affect satellite cell activation and proliferation. A previous study has found that muscle stem cells (MuSCs) attract capillary ECs by secreting VEGFA, and as a response, the ECs help maintain MuSCs quiescence through Dll4 [36]. Moreover, the interplay between satellite cells and the vasculature is bidirectional [32, 35]. Activated satellite cells can secrete factors such as VEGFs to promote angiogenesis [37], thereby enhancing blood supply to the repair site [38]. This reciprocal relationship ensures efficient muscle regeneration by coordinating cellular and vascular growth [32, 35]. In this study, mRNA sequencing and qPCR results demonstrated that both MFG and DCA treatments upregulated *VEGFs* expression, indicating improvement of angiogenesis in skeletal muscles and enhancement of muscle development and repair. Additional experiments are required to further explore the roles of the satellite cell niche and angiogenesis.

DCA is a secondary bile acid, and TGR5 is one of its important receptors [39]. TGR5 is expressed in various tissues, including brown adipose tissue, small intestine and skeletal muscles [40, 41]. When bound to its ligands, TGR5 induces intracellular cAMP production, activating the PKA/cAMP/pCREB pathway [42], which regulates numerous biological processes [43]. Previous research has reported that the PKA/pCREB pathway is involved in VEGFs expression in mouse macrophages and human microvascular endothelial cell line‐1 (HMEC‐1) cells [25, 26]. In this study, DCA improved muscle angiogenesis by activating TGR5/cAMP/PKA/pCREB/VEGFA to enhance muscle development. A previous study has found that increasing the expression of TGR5 improves muscle function [44]. Conversely, some other studies discovered that deletion of TGR5 in muscle fibres prevented skeletal muscle growth inhibition caused by CA and DCA. These bile acids induce muscle growth inhibition through the TGR5 receptor [45]. These findings together indicate that bile acids have varying effects on skeletal muscles depending on the environment.

In conclusion, PR and MFG promote muscle angiogenesis to counteract the DEX‐induced muscle growth inhibition by activating the TGR5/cAMP/PKA/pCREB/VEGFs axis mediated by *C. aerofaciens‐*derived DCA.

## Conflicts of Interest

The authors declare no conflicts of interest.

## Supporting information


**Table S1.** qPCR primer sequence.
**Figure S1.** Effects of AEPR and MFG on food intake and liver in mice.
**Figure S2.** MFG promotes muscle regeneration.
**Figure S3.** Effects of MFG under DEX treatment in food intake and tissues.
**Figure S4.** Administration of fructose or MFG (0.5 g/Kg body weight) alleviated the glucocorticoid‐induced growth inhibition in muscle development.
**Figure S5.** Effects of AEPR and fructose on CD31 expression.
**Figure S6.** Effects of DCA on food intake, fat/liver mass and CD31 expression and effects of DEX and MFG on bile acid synthesis in liver.
