# Correction to ‘*Polygonati Rhizoma* Prevents Glucocorticoid‐Induced Growth Inhibition of Muscle via Promoting Muscle Angiogenesis Through Deoxycholic Acid’

**DOI:** 10.1002/jcsm.70053

**Published:** 2025-09-10

Shi S , Li R , Han Y , Xie J , Wang S , Liu J , Wan F , Hou G , Liu Z , Sun X , Zuo B , Jia Z , Mei Z , Song T . 
*Polygonati Rhizoma* Prevents Glucocorticoid‐Induced Growth Inhibition of Muscle via Promoting Muscle Angiogenesis Through Deoxycholic Acid. J Cachexia Sarcopenia Muscle. 2025 Jun;16(3):e13853. 10.1002/jcsm.13853.40522031 PMC12168230

In Methods section ‘2.12 Cultivation of *Collinsella aerofaciens*’, the sentence ‘ *C. aerofaciens* was previously isolated in our lab’. should read as:

The *C. aerofaciens* strain used in this study was provided by Dr. Qingbiao Xu from Huazhong Agricultural University.

In Acknowledgements section, the sentence: ‘The authors would like to thank all the members of the Adipo‐mystery (ADM) group, Dr. Qingbiao Xu and Ms. Wenjuan Du from Huazhong Agricultural University’. should read as:

The authors would like to thank all the members of the Adipo‐mystery (ADM) group from Huazhong Agricultural University.

We greatly appreciate the kindly help from Dr. Qingbiao Xu and we have discussed with him. It would be better to remove the name ‘Dr. Qingbiao Xu and Ms. Wenjuan Du’ from acknowledgements section and revise the description in methods section.

These changes are important for the accuracy and completeness of the manuscript record and will not affect the overall conclusion of the article.

We apologize for this error.

