# Supplementary material for: Polygonati Rhizoma Prevents Glucocorticoid‐Induced Growth Inhibition of Muscle via Promoting Muscle Angiogenesis Through Deoxycholic Acid
Source: J Cachexia Sarcopenia Muscle. 2025 Jun 16;16(3):e13853. doi: 10.1002/jcsm.13853 (PMC12168230; doi:10.1002/jcsm.13853)
Supplement: Supplementary file 1 — Table S1. qPCR primer sequence. Figure S1. Effects of AEPR and MFG on food intake and liver in mice. Figure S2. MFG promotes muscle regeneration. Figure S3. Effects of MFG under DEX treatment in food intake and tissues. Figure S4. Administration of fructose or MFG (0.5 g/Kg body weight) alleviated the glucocorticoid‐induced growth inhibition in muscle development. Figure S5. Effects of AEPR and fructose on CD31 expression. Figure S6. Effects of DCA on food intake, fat/liver mass and CD31 expression and effects of DEX and MFG on bile acid synthesis in liver. [file JCSM-16-e13853-s001.docx]

**Supplemental methods**

**Gavage of glucose, fructose or MFG in DEX-induced muscle growth inhibition mouse model**

Forty male C57BL/6 mice at three weeks of age were randomly assigned to five groups, with each group consisting of seven mice (n = 8). The five groups are as follows: CON group: The mice received intraperitoneal injection of PEG 400 solution with an equivalent volume to that of the DEX group; DEX group: The mice received intraperitoneal injection of DEX (10 mg/kg/day) for a duration of two weeks; DEX-FRU group: The mice were intraperitoneal injected with DEX (10 mg/kg/day) for two weeks, followed by gavaged with fructose at a dose of 0.5 g/kg body weight by gavage for another two weeks; DEX-GLU group: The mice were intraperitoneal injected with DEX (10 mg/kg/day) for two weeks, followed by gavaged with glucose at a dose of 0.5 g/kg body weight by gavage for another two weeks; DEX-MFG group: The mice were intraperitoneal injected with DEX (10 mg/kg/day) for two weeks, followed by gavaged with MFG (a mixture of fructose and glucose at the ratio of 1: 1) at a dose of 0.5 g/kg body weight by gavage for another two weeks. Subsequently, the mice were sacrificed, and samples were collected for further testing.

The preparation method for the dexamethasone solution is as follows: Initially, dexamethasone weighing 0.5 g, measured with precision using an analytical balance, is solubilized in 1 milliliter of dimethyl sulfoxide (DMSO) to yield a concentrated stock solution of 500 mg/mL. Subsequently, an aliquot of 500 µL from this stock is admixed with 25 mL of polyethylene glycol 400 (PEG400). Upon achieving complete dissolution, the mixture is further diluted with 24.5 mL of physiological saline solution. This serial dilution results in a 100-fold dilution factor, culminating in the formulation of a working solution with a final concentration of 5 mg/mL.

The preparation method for fructose solution is as follows: 1 g fructose is mixed and dissolved in 20 mL of water.

The preparation method for glucose solution is as follows: 1 g glucose is mixed and dissolved in 20 mL of water.

The preparation method for MFG solution is as follows: 0.25 g of glucose and 0.25 g of fructose are mixed and dissolved in 20 mL of water.

**Bodipy dye staining of liver**

DAPI (blue) staining was used for nucleus and bodipy (green) staining was used for lipid droplets in liver tissue. For bodipy staining, frozen slides of liver tissue were washed with PBS and then stained with BODIPY 493/503 (D3922, Thermo Fisher Scientific, USA) for 15 min in the dark at 37 °C. Slides were then washed twice with PBS for 5 min each and stained with DAPI. Finally, the samples were covered with a fluorescent mounting medium to prepare for imaging.

**Measurement of** **triglycerides, total cholesterol, and uric acid**

A fully automatic biochemistry analyzer (Mindrayanimal, Shenzhen, China) was utilized to measure the triglycerides, total cholesterol and uric acid in serum. Hepatic triglycerides and total cholesterol were measured by triglyceride assay kit (A110-1-1, Jiancheng, China) and total cholesterol assay kit (A111-1-1, Jiancheng, China).

**Measurement of ALT, AST, Mb and cTnI**

Serum ALT and AST were measured by ALT assay kit (C009-2-1, Jiancheng, China) and AST assay kit (C010-2-1, Jiancheng, China). Serum Mb and cTnI were measured by Mb ELISA kit (MM-0518M2, Meimian, China) and cTnI ELISA kit (MM-0379M1, Meimian, China).

**Supplemental table and figures**

**Table S1. qPCR primer sequence.**

| Specie | *Gene* | Forward primer (5’-3’) | Reverse primer (5’-3’) | Reference |
| --- | --- | --- | --- | --- |
| Mouse | *CD31* | AGTCAGAGTCTTCCTTGCCC | TCTGTTTGGCCTTGGCTTTC | ^1^ |
|  | *VEGFA* | TGGACCCTGGCTTTACTGCT | GCAGTAGCTTCGCTGGTAGA | ^2^ |
|  | *VEGFD* | CTCCAGGAACCCACTCTCTG | TCCTGGCTGTAGAGTCCCTG | ^3^ |
|  | *VEGFR1* | GGCCCGGGATATTTATAAGAAC | CCATCCATTTTAGGGGAAGTC | ^4^ |
|  | *VEGFR2* | ACGAGGAGAGAGGGTCATCT | CAACAGGGACACACTCTCCT | ^1^ |
|  | *TGR5* | GAGCGTCGCCCACCACTAGG | CGCTGATCACCCAGCCCCATG | ^5^ |
|  | *MYH11* | ACAGGAGGCCAGAGAGAAAC | GTACTGCTCTGCCATCTTGC | ^1^ |
|  | *TAGLN* | GGTGTGGCTGAAGAATGGTG | TTGAGCCACCTGTTCCATCT | ^1^ |
|  | *ACTA2* | GCTATTCAGGCTGTGCTGTC | GGTAGTCGGTGAGATCTCGG | ^1^ |
|  | *SMTN* | CCTCCCACAAGAAGCAGAGA | ATTCTGCTCTCTGGTTGCCT | ^1^ |
|  | *Cyp7a1* | AGCAACTAAACAACCTGCCAGTACTA | GTCCGGATATTCAAGGATGCA | ^6^ |
|  | *Cyp8b1* | GAAGATCCACCACTACAGCAT | GGACAAAGGTCTTCATCTCG | ^6^ |
|  | *Cyp27a1* | CCAGGCACAGGAGAGTACG | GGGCAAGTGCAGCACATAG | ^6^ |
|  | *Cyp7b1* | GAAAACTCTTCAAAGGCAACATGG | ACTGGAAAGGGTTCAGAACAAATG | ^6^ |
|  | *FASN* | CCAAGCAGGCACACACAA | CACTCACACCCACCCAGA | ^7^ |
|  | *SREBP1* | TGACCCGGCTATTCCGTGA | CTGGGCTGAGCAATACAGTTC | ^7^ |
|  | *GAPDH* | GGGTGTGAACCACGAGAAAT | CCTTCCACAATGCCAAAGTT | ^8^ |

**
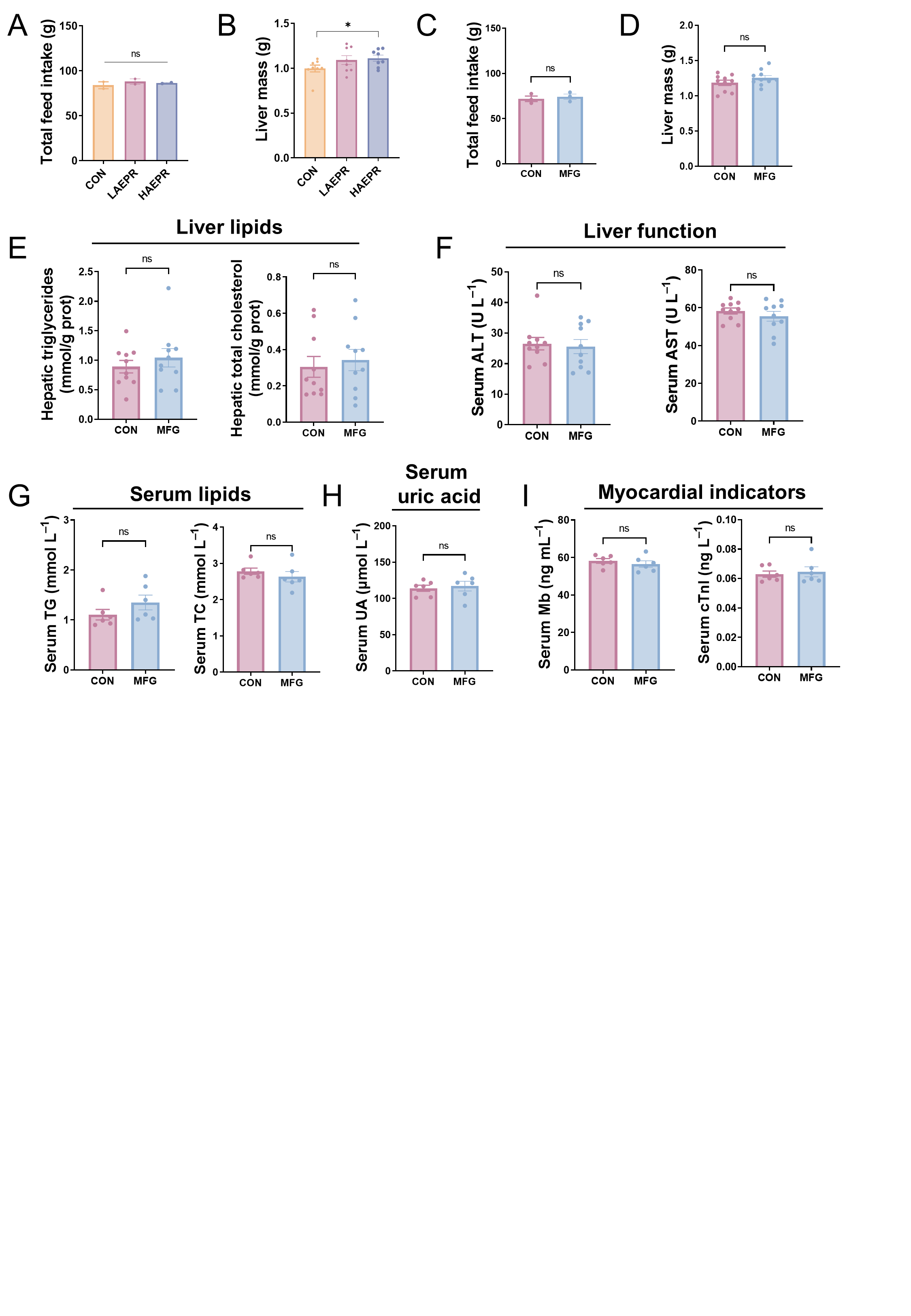
 Fig. S1. Effects of AEPR and MFG on food intake and liver in mice**

(A) Total feed intake in AEPR treatment; (B) Liver mass in AEPR treatment; (C) Total feed intake in MFG treatment; (D) Liver mass in MFG treatment; (E) Hepatic triglycerides and total cholesterol in MFG treatment; (F) Serum ALT and AST in MFG treatment; (G) Serum TG and TC in MFG treatment; (H) Serum uric acid in MFG treatment; (I) Serum Mb and cTnI in MFG treatment**.** Data information: t test was used in this figure where error bars represent SD, and **P* < 0.05.


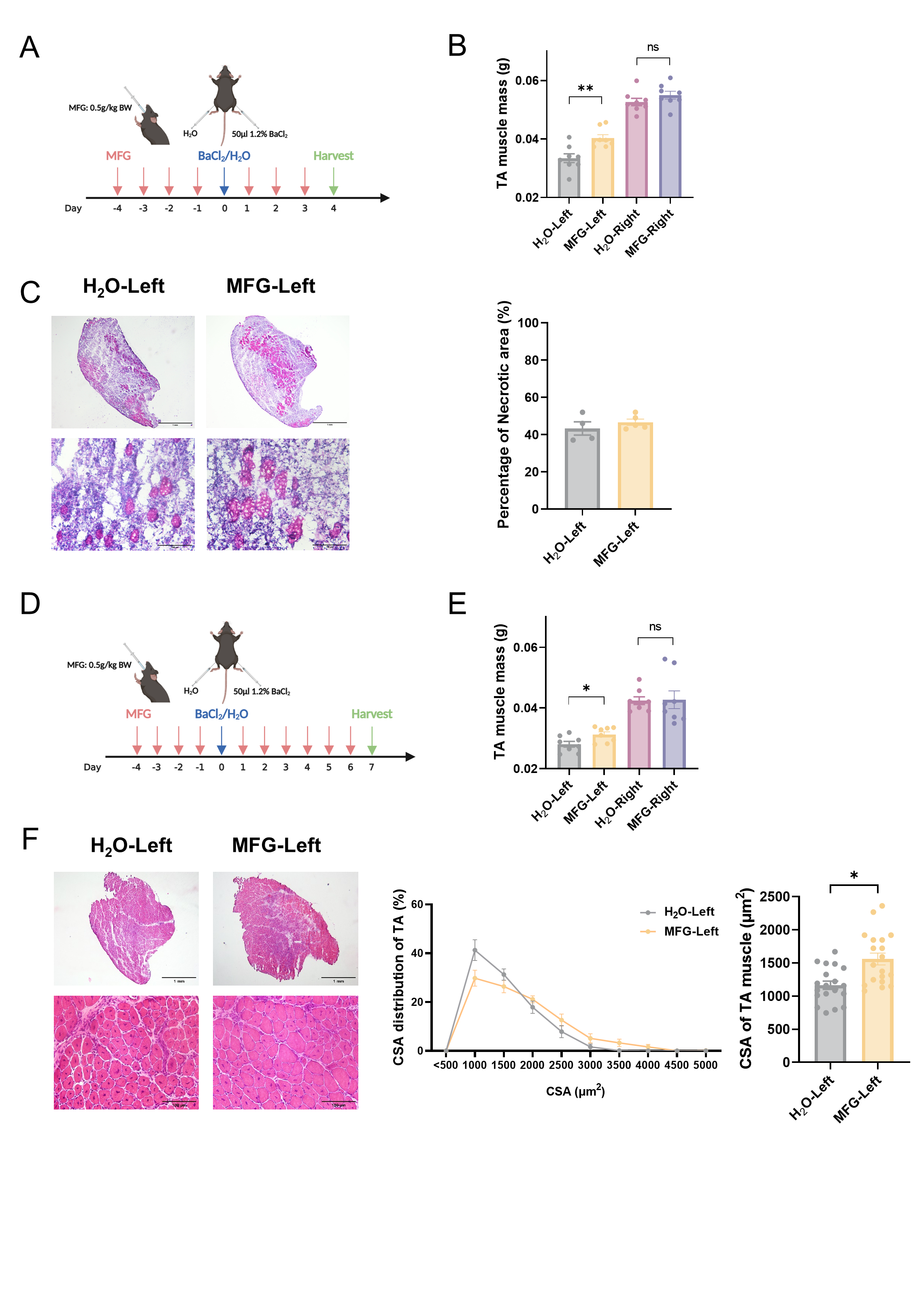


**Fig. S2. MFG promotes muscle regeneration**

(A) Study design of the BaCl_2_-induced muscle injury model with MFG treatment for 4 days, n = 8 biological replicates in each group; (B and C) TA muscle mass and representative H&E staining of myofiber cross section of TA with percentage of necrotic area; (D) Study design of the BaCl_2_-induced muscle injury model with MFG treatment for 7 days, n = 8 biological replicates in each group; (E and F) TA muscle mass and representative H&E staining of myofiber cross section of TA with CSA distribution and average CSA of muscle fiber. Data information: t test was used in this figure where error bars represent SD, and **P* < 0.05, ***P* < 0.01.


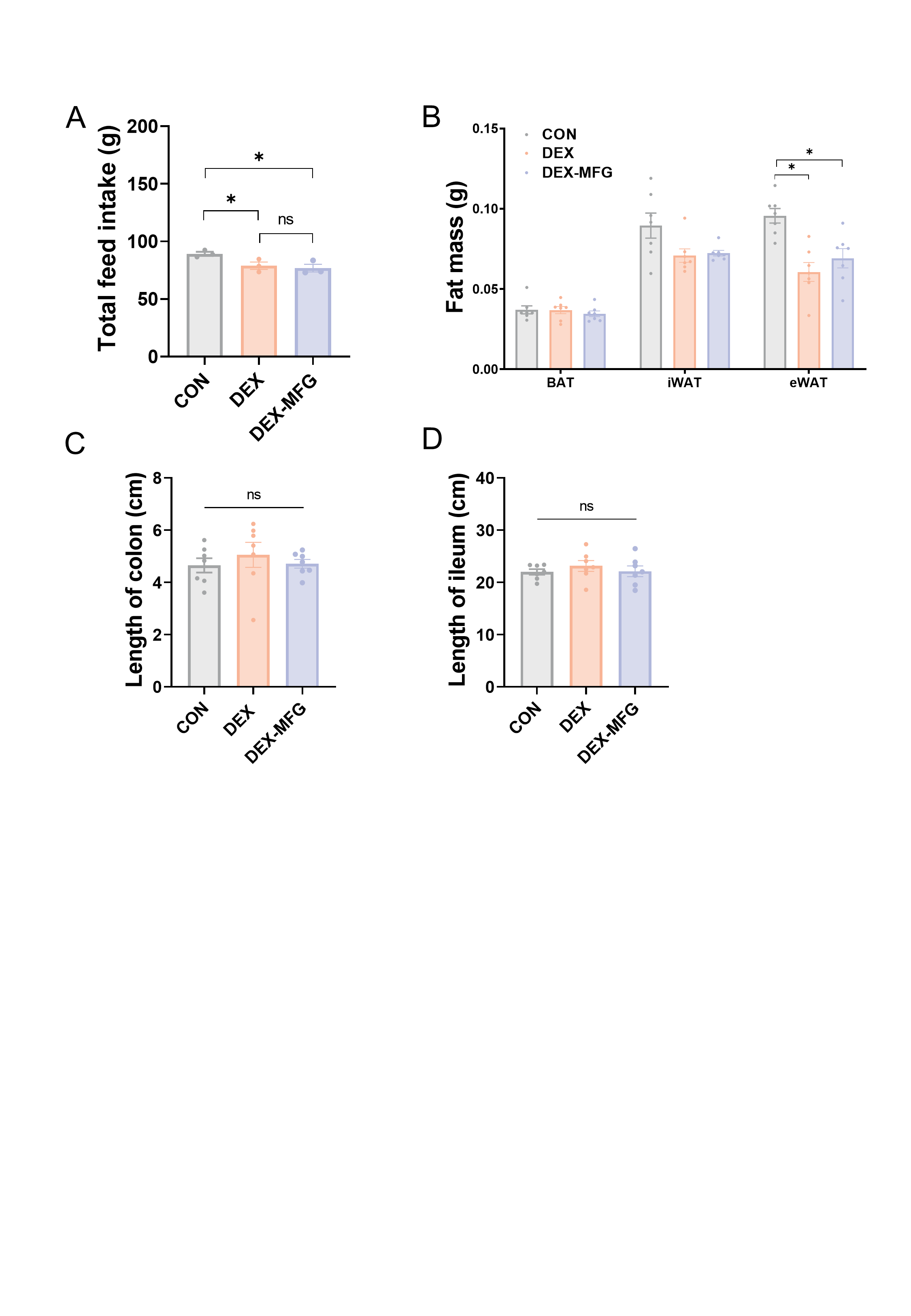


**Fig. S3. Effects of MFG under DEX treatment in food intake and tissues**

(A) Total feed intake; (B) Adipose tissues mass; (C) Length of colon; (D) Length of ileum. Data information: t test was used in this figure where error bars represent SD, and **P* < 0.05.


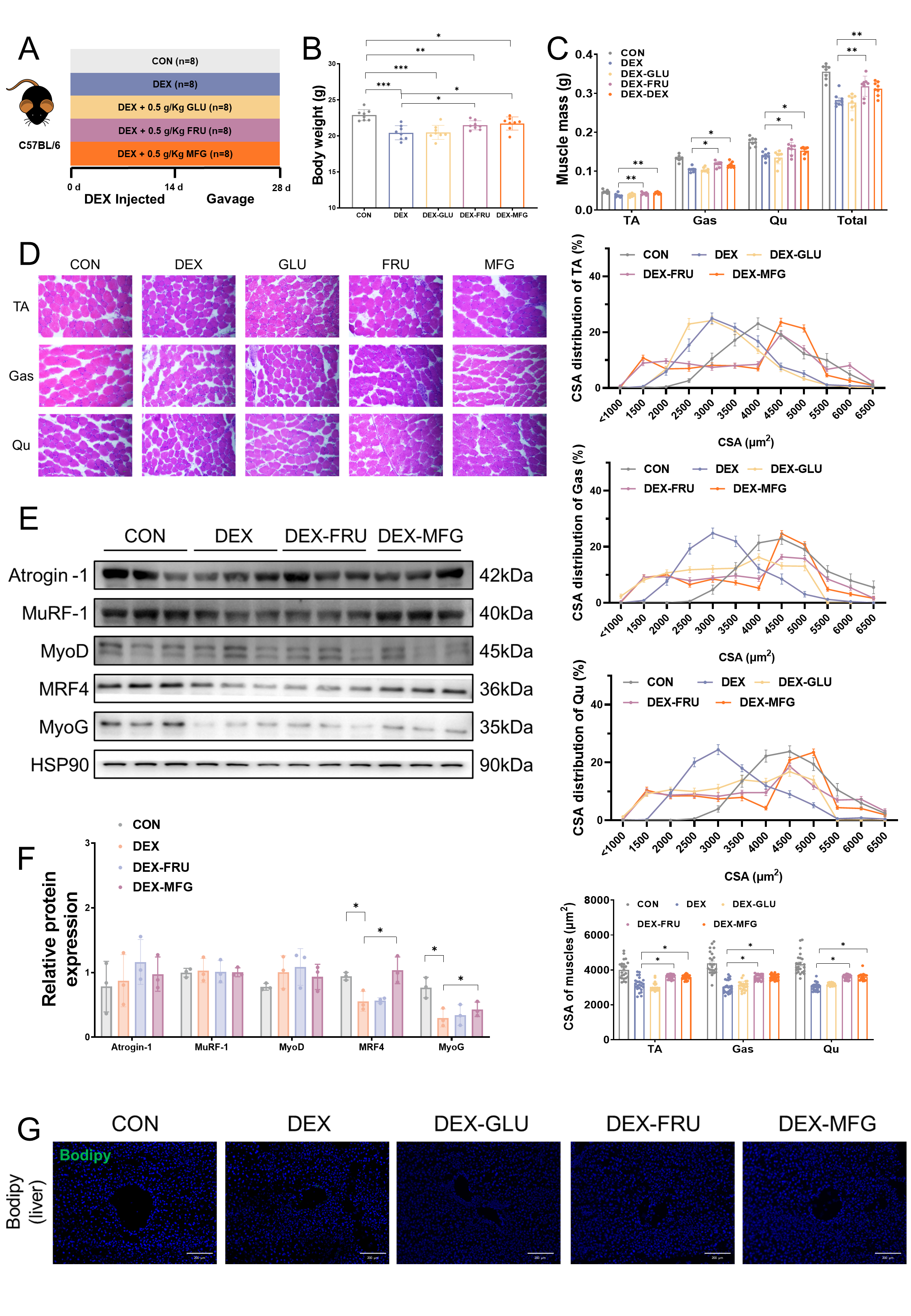


**Fig. S4. Administration of fructose or MFG (0.5 g/Kg body weight) alleviated the glucocorticoid-induced growth inhibition in muscle development**

(A) Study design, n = 8 biological replicates in each group; (B) Body weight under different treatments; (C) Muscle mass (D) Representative H&E staining of myofiber cross-section of TA, Gas and Qu and CSA distribution and average CSA of muscle fiber; (E and F) Western blot and quantification of MyoD, MyoG, MRF4, Atrogin-1 and MuRF-1; (G) Representative images of bodipy immunofluorescence staining in liver. Data information: t test was used in this figure where error bars represent SD, and **P* < 0.05; ***P* < 0.01; ****P* < 0.001.


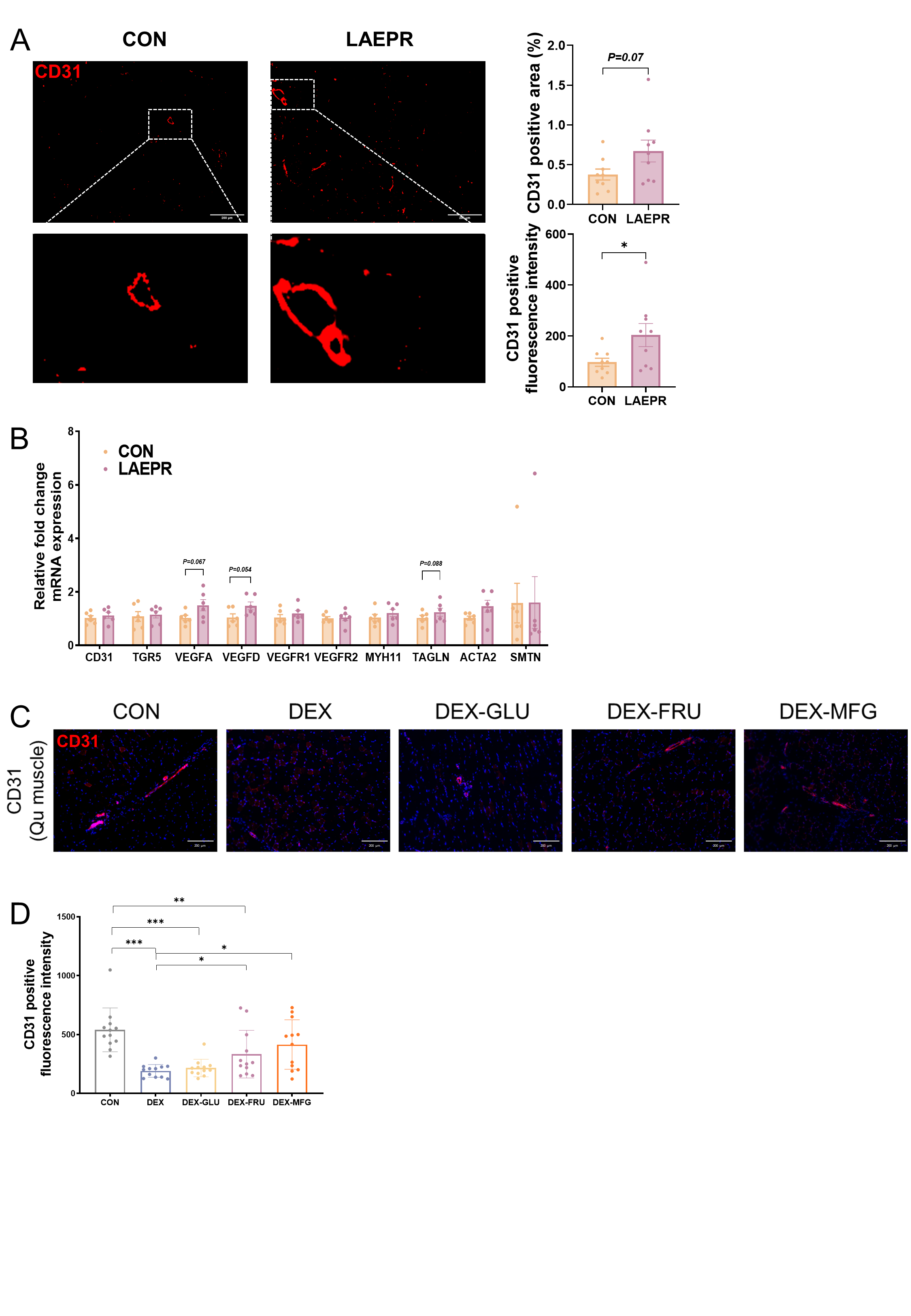


**Fig. S5. Effects of AEPR and fructose on CD31 expression**

(A) Representative images and quantification of CD31 immunofluorescence staining in Qu muscle of LAEPR treatment group; (B) mRNA levels of angiogenesis and contractility genes in LAEPR treatment group; (C and D) Representative images and quantification of CD31 immunofluorescence staining in Qu muscle. Data information: t test was used in this figure where error bars represent SD, and **P* < 0.05; ***P* < 0.01; ****P* < 0.001.


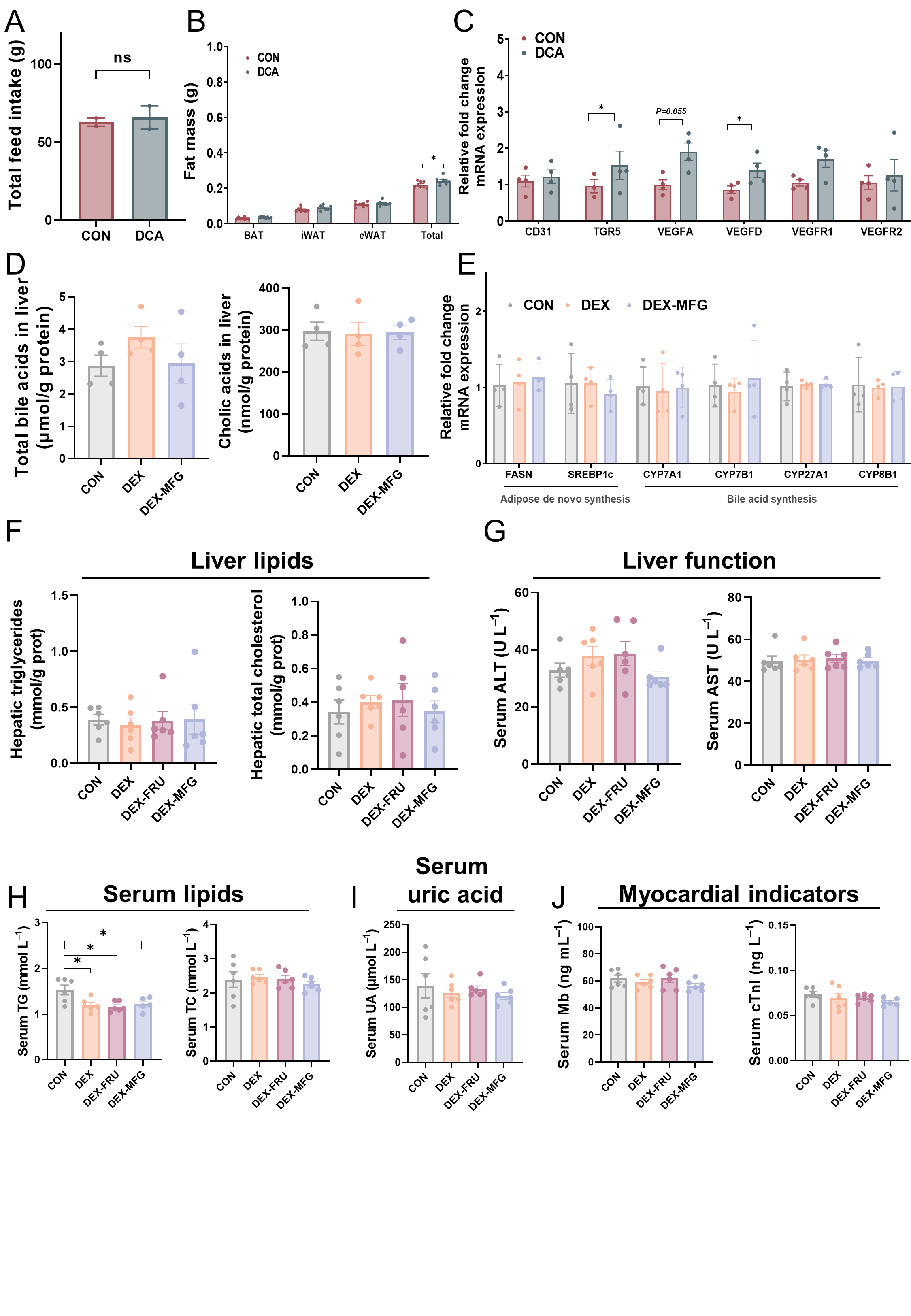


**Fig. S6. Effects of DCA on food intake, fat/liver mass and CD31 expression and** **effects of DEX and MFG on bile acid synthesis in liver**

(A) Total feed intake in DCA treatment; (B) Fat mass in DCA treatment; (C) mRNA levels of angiogenesis genes in DCA group; (D) Total bile acids in liver; (E) Cholic acids in liver; (F) Hepatic triglycerides and total cholesterol in MFG treatment; (G) Serum ALT and AST in MFG treatment; (H) Serum TG and TC in MFG treatment; (I) Serum uric acid in MFG treatment; (J) Serum Mb and cTnI in MFG treatment**.** Data information: t test was used in this figure where error bars represent SD, and **P* < 0.05.

**References**

1. Yang J, Xu G, Xu Y, Luo P, Yuan Y, Yao L, Zhou J, Zhu Y, Gyawali I, Xu C, Feng J, Ma Z, Zeng Y, Wang S, Gao P, Zhu C, Jiang Q and Shu G. AKG/OXGR1 promotes skeletal muscle blood flow and metabolism by relaxing vascular smooth muscle. *Life Metabolism*. 2022;1:285-297.

2. Wang X-H, Ao Q-G and Cheng Q-L. Caloric restriction inhibits renal artery ageing by reducing endothelin-1 expression. *Annals of Translational Medicine*. 2021;9.

3. Deckers MM, Karperien M, van der Bent C, Yamashita T, Papapoulos SE and Löwik CW. Expression of vascular endothelial growth factors and their receptors during osteoblast differentiation. *Endocrinology*. 2000;141:1667-1674.

4. Duan J-L, Zhou Z-Y, Ruan B, Fang Z-Q, Ding J, Liu J-J, Song P, Xu H, Xu C and Yue Z-S. Notch-Regulated c-Kit–Positive Liver Sinusoidal Endothelial Cells Contribute to Liver Zonation and Regeneration. *Cellular and molecular gastroenterology and hepatology*. 2022;13:1741-1756.

5. Kumar DP, Rajagopal S, Mahavadi S, Mirshahi F, Grider JR, Murthy KS and Sanyal AJ. Activation of transmembrane bile acid receptor TGR5 stimulates insulin secretion in pancreatic β cells. *Biochemical and biophysical research communications*. 2012;427:600-605.

6. Hashimoto M, Kobayashi K, Watanabe M, Kazuki Y, Takehara S, Inaba A, Nitta S-i, Senda N, Oshimura M and Chiba K. Knockout of mouse <em>Cyp3a</em> gene enhances synthesis of cholesterol and bile acid in the liver<sup></sup>. *Journal of Lipid Research*. 2013;54:2060-2068.

7. Zhang C, Hu J, Sheng L, Yuan M, Wu Y, Chen L, Wang G and Qiu Z. Ellagic acid ameliorates AKT-driven hepatic steatosis in mice by suppressing de novo lipogenesis via the AKT/SREBP-1/FASN pathway. *Food & Function*. 2019;10:3410-3420.

8. Xie J, Peng C, Zhao Q, Wang X, Yuan H, Yang L, Li K, Lou X and Zhang Y. Osteogenic differentiation and bone regeneration of iPSC-MSCs supported by a biomimetic nanofibrous scaffold. *Acta biomaterialia*. 2016;29:365-379.
